# RNASeq analysis of giant cane reveals the leaf transcriptome dynamics under long-term salt stress

**DOI:** 10.1186/s12870-019-1964-y

**Published:** 2019-08-15

**Authors:** Angelo Sicilia, Giorgio Testa, Danilo Fabrizio Santoro, Salvatore Luciano Cosentino, Angela Roberta Lo Piero

**Affiliations:** Department of Agriculture, Food and Environment, University of Catania, Via Santa Sofia 98, 95123 Catania, Italy

**Keywords:** Bioenergy crops, De novo assembly, Giant reed, Leaf transcriptome, RNA-seq, Salt stress

## Abstract

**Background:**

To compensate for the lack of information about the molecular mechanism involved in *Arundo donax* L. response to salt stress, we de novo sequenced, assembled and analyzed the *A. donax* leaf transcriptome subjected to two levels of long-term salt stress (namely, S3 severe and S4 extreme).

**Results:**

The picture that emerges from the identification of differentially expressed genes is consistent with a salt dose-dependent response. Hence, a deeper re-programming of the gene expression occurs in those plants grew at extreme salt level than in those subjected to severe salt stress, probably representing for them an “*emergency*” state. In particular, we analyzed clusters related to salt sensory and signaling, transcription factors, hormone regulation, Reactive Oxygen Species (ROS) scavenging, osmolyte biosynthesis and biomass production, all of them showing different regulation either versus untreated plants or between the two treatments. Importantly, the photosynthesis is strongly impaired in samples treated with both levels of salinity stress. However, in extreme salt conditions, a dramatic switch from C3 Calvin cycle to C4 photosynthesis is likely to occur, this probably being the more impressive finding of our work.

**Conclusions:**

Considered the distinct response to salt doses, genes either involved in severe or in extreme salt response could constitute useful markers of the physiological status of *A. donax* to deepen our understanding of its biology and productivity in salinized soil. Finally, many of the unigenes identified in the present study have the potential to be used for the development of *A. donax* varieties with improved productivity and stress tolerance, in particular the knock out of the GTL1 gene acting as negative regulator of water use efficiency has been proposed as good target for genome editing.

**Electronic supplementary material:**

The online version of this article (10.1186/s12870-019-1964-y) contains supplementary material, which is available to authorized users.

## Background

By 2050, human population may grow to 9.6 billion or about 2.0 people ha^− 1^ of cultivated land, which calls for considerable increases in agricultural production [1]. To meet growing world population and improvements in nutrition and food quality, the demand for energy, particularly with respect to terrestrial and air transports, will also increase. Since petro-chemical resources will become less available, an increase demand for alternative energy that substitutes for fossil fuel transport energy will be met in significant measure by biofuels [2, 3]. Therefore, the end use of land as a global resource is likely to become the focus of intensified competition between food or feed function and biofuel cultivation. At present, the main feedstock of biofuels all over the world is agricultural product such as sugarcane, cassava, wheat, potato and other crops called *first generation biofuels* [4]*.* Cellulosic crops (traditionally called energy crops), referred to as *second generation biofuels*, cultivated with the specific purpose of producing alternative fuels might represent a promising alternative considering that they may satisfy at least part of the energy demand and at the same time mitigate greenhouse gases emission (GHGs), in particular the carbon dioxide emission, by sequestering them into biomass [5]. As cellulosic feedstocks cannot be produced on arable lands due to the aforementioned environmental and economic concerns, a recommended strategy is to grow them on “marginal lands”, usually described as unproductive lands, due to poor soil properties, bad quality of underground water, drought or unfavorable climatic conditions, subsequently with no or little potential of profitability for conventional food crops [6]. Salt affected soils are also considered marginal due to high salinity and sodicity [7]. They may be practically suitable to grow perennial rhizomatous grasses, which are better adapted to poor soils providing high cellulosic biomass without competition with food crops and overcoming risks for food security. Perennial rhizomatous grasses also display several positive attributes because of their low demand for nutrient inputs consequent to the recycling of nutrients by their rhizomes, and resistance to biotic and abiotic stresses [8]. Several studies have been conducted all over the world to evaluate the potential of marginal lands for selected plant species for biomass–bioenergy production. The European project, Optimization of Perennial Grasses for Biomass Production in the Mediterranean Area (OPTIMA) was launched with the aim to establish new strategies for the sustainable use of the marginal land in Mediterranean areas [9]. In that project, *Arundo donax* L. (giant reed), among others perennial grasses, was studied in sufficient depth, in terms of biomass production potential in warm-temperate and semi-arid areas [9, 10]. The adaptability of the plants to different kinds of environments, soils and growing conditions, in combination with the high biomass production confers on *A. donax* many advantages when compared to other energy crops. *A. donax* requires low irrigation and nitrogen inputs and salinity does not seem to affect plant growth, as it is possible to achieve acceptable biomass yields under high salinity conditions due to its halophyte behavior [11]. *Arundo donax* L., common name “giant cane” or “giant reed”, is a polyploid perennial grass plant belonging to the *Poaceae* family. The phylogenetic origin is unclear, although recent evidences obtained by chloroplastic DNA sequencing suggest a middle-east origin [12]. *A. donax* is a sterile plant because of the defective development of male and female gametophytes [13]. The reproduction only occurs by the vegetative growth of rhizomes and of stem nodes of broken canes. Consequently, the genotypic diversity among clonal populations is expected to be very low and the genetic improvement of this plant to ameliorate its performance as energy crop in adverse environmental conditions is mainly based on clonal selection [14, 15]. As transformation and regeneration protocols are available [16, 17], the genetic engineering could represent a feasible option for the improvement of *A. donax* and these approaches might greatly take advantage from the availability of transcriptomic data sets. Next-generation high-throughput sequencing techniques have become an increasingly useful tool for exploring whole plant genomes, providing a means for analyzing plant molecular regulatory mechanisms in specific environments such as various abiotic stress conditions, including heavy metal toxicity, herbicide toxicity and salt toxicity [18–20]. Soil salinization is referred as the accumulation of soluble salts in the soils [21]. This takes place particularly in arid and semi-arid areas characterized by both great amount of evaporation and minimal precipitation volumes. Salinity affects all plant physiological responses and production by reducing the uptake of water and nutrients and creating an ion imbalance and toxicity [22–24]. Salt may affect plant growth indirectly by decreasing the rate of photosynthesis and stomatal conductance. Stomatal closure is considered as the most dramatic response that occurs in plants after exposure to salinity owing to the osmotic effect of salt outside the roots. Moreover, the reduced rate of photosynthesis increases the formation of reactive oxygen species (ROS) leading to oxidative stress [22–25]. The hormone-mediated regulatory network is a key molecular mechanism of salt tolerance in various plants, and transcriptome analysis has indicated that abscisic acid (ABA) signaling plays an important role [26]. After signal perception and transmission, plants respond to salinity by coordinating the regulation of gene expression and triggering a series of physiological and biochemical changes to adapt to high-salt environments including the activation of specific transcription factors (TFs), and the control of downstream structural genes [23, 27]. In addition, the synthesis of osmolytes such as proline, betaine, mannitol, flavonoids, and organic acids in plants increases, and related synthetic genes are up-regulated [28, 29]. Nevertheless, salt stress response mechanisms in plants remain poorly understood due to the complexity of the response process and the genetic variability among plant species. Moreover, our knowledge of the genetic bases of salt tolerance is largely based on genetic studies in model or crop species [24]. De novo RNA sequencing (RNA-Seq) assembly might facilitate the study of transcriptomes for non-model plant species for which the genome sequence is not available by enabling an almost exhaustive survey of their transcriptomes and allowing the discovery of virtually all expressed genes in a plant tissue under abiotic stress. Useful *A. donax* genomic resources were provided by the work of Sablok et al. [30] which used tissue-specific NGS of four different organs (leaf, culm, bud and root) of one *A. donax* ecotype constituting a comprehensive reference catalog of transcripts aimed at characterizing and improving the spatial and temporal patterns of expression underlying the high productivity of biomass. Moreover, a shoot transcriptome was obtained from an *A. donax* invasive ecotype [31]. To provide a more complete gene expression catalogue and allow a comprehensive comparison among various assemblies, a genomic resource was generated using three ecotypes originating from distant geographical locations that, for this reason, could have accumulated heritable phenotypic differences [32]. Recently, the characterization of *A. donax* transcriptome in response to drought has been reported leading to the identification of early-responsive genes to water stress which might constitute a basin of information for the improvement of giant reed productivity under water limitation [33]. Considering the frequent occurrence of soil salinity in the Mediterranean area and the potential use of marginal soil for energy crop cultivation aimed to overcome the incoming food security risks, a deep knowledge of the global transcriptomic response of giant reed to salt is needed seeing as it is not yet available. In this study, we analyzed the effect of two levels of prolonged period of salt stress upon the *A. donax* whole leaf transcriptome by using a RNA-seq approach in order to elucidating the biological processes underlying the salt tolerance in a non- model plant. This study lays the foundation to select candidate genes for cis- but also trans-genesis with the aim to develop plants with improved salt stress tolerance.

## Results

### Effect of salt stress upon *A. donax* morpho-biophysiological parameters

As described in the Methods section, *A. donax* G2, G18 and G20 morpho-biometric and physiological parameters were measured at sampling date after being subjected to two levels of prolonged salt stress imposition (S3, severe and S4, extreme), being both doses much higher than that used to define a soil area as “salinized” (EC 4 dS m^− 1^). Considering the average values of the three clones, we observed that both the leaf number per pot, the stem number and the main stem height per pot, were significantly reduced by salt stress, and this effect was more pronounced in correspondence of the S4 treated samples (Additional file 1: Figure S1a, S1b and S1c). Similarly, physiological parameters such as SPAD, leaf chlorophyll content, net photosynthesis and biomass yield per pot decreased especially under extreme salt stress conditions (S4) compared with untreated S0 samples (Additional file 1: Figure S1d, S1e and S1f). The ecotypes under investigation exhibited different phenotypes in response to salt treatments, although they reproduce asexually and, for this reason, they should have low levels of genetic diversity. In particular, the whole data analysis revealed that G20 clone did not growth under extreme salt stress conditions, suggesting that it is highly sensitive to high salt concentration (Additional file 1: Figure S1). Although both G2 and G18 clone grew under severe (S3) and extreme (S4) salt stress conditions, G2 clone showed higher stem and leaf number per pot, and higher physiological parameters than those recorded in G18 clone in S4 conditions. Indeed, G2 clone produced considerable higher biomass yield than that reported in G18 clone in extreme salt stress conditions, in an environment in which likely none crops could have survived [34] (Additional file 1: Figure S1f). Therefore, further transcriptomic analysis was conducted upon G2 clone subjected to severe and extreme salt stress. The picture of giant reed phenotype under salt stress is shown in Additional file 2: Figure S2.

### Transcript assembly and annotation

**Fig. 1:**
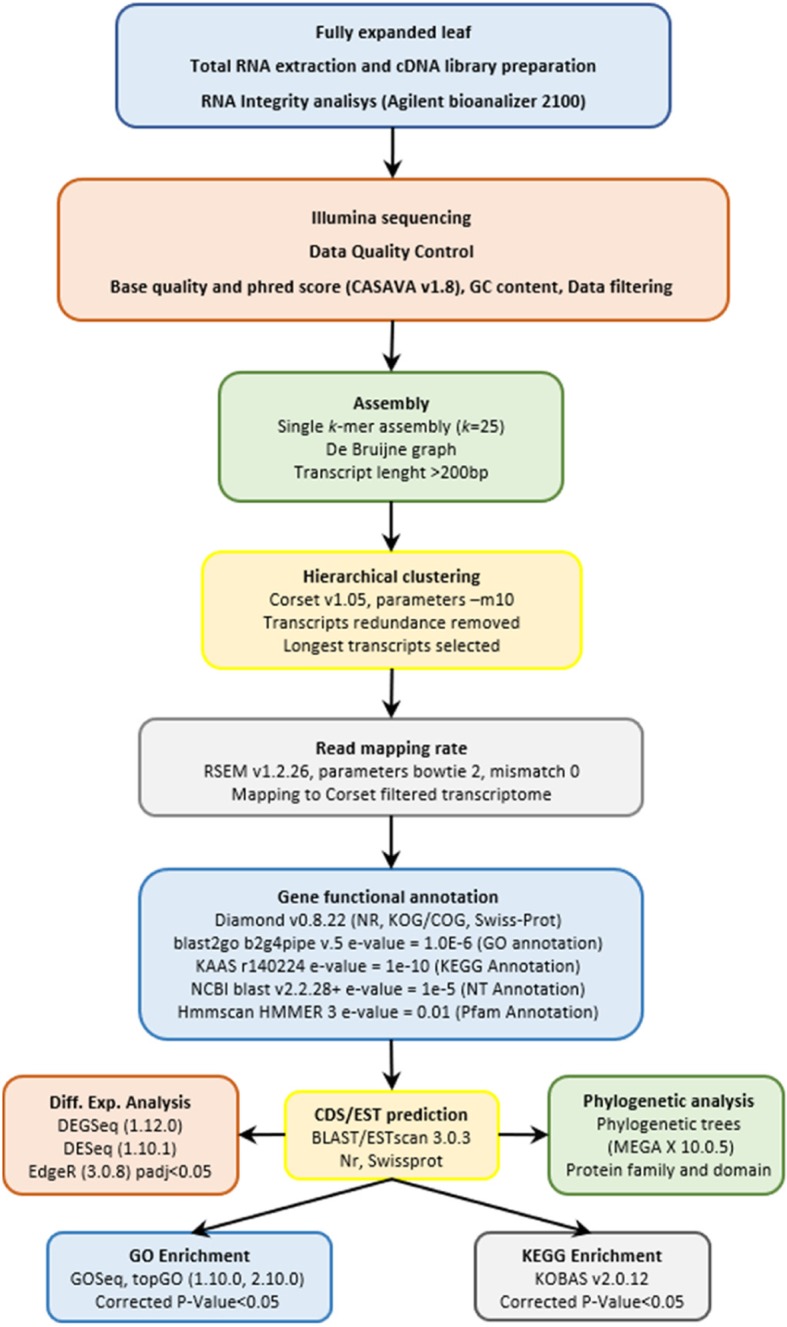
Flowchart of de novo assembly and analysis of *Arundo donax* leaf transcriptome. After the fully expanded leaf sampling, total RNA extraction and cDNA library preparation was carried out. The RNA integrity and quality analysis were performed (blue) before the Illumina sequencing. The sequencing output data were subjected to quality control of both reads and bases and data filtering (orange) in order to remove containing adapter reads or reads of low quality. Clean data were used for the de novo assembly of transcripts choosing the single k-mer approach (k = 25) (green), and the pre-assembled transcriptome obtained was further processed with Corset for hierarchical clustering by removing transcripts redundancy and by selecting the longest transcripts as unigenes (yellow). The quality of the assembly was assessed by mapping back the reads onto the filtered transcriptome (grey). Finally, gene functional annotation, CDS/EST prediction, differential expression analysis, phylogenetic analysis and GO and KEGG enrichment were carried out

In this study, we carried out a comprehensive identification of transcriptional responses of *A. donax* G2 clone to two different levels of prolonged salt stress by RNA-Seq (see experimental design in Methods section). In Fig. 1, a flow chart for de novo transcriptome assembly is reported (see details in Fig.1 caption). Raw reads were filtered to remove reads containing adapters or reads of low quality, so that the downstream analyses are based on a total of 643 million clean reads with an average of ~ 71.5 million reads (~ 10.7 G) per sample, the average percentage of Q30 and GC being 94.7 and 53.3%, respectively (Table 1). De novo assembly of clean reads resulted in 255,809 transcripts and 186,740 unigenes with N50 length of 1857 and 1845, respectively (Table 1), in line with previous N50 reports [30, 33], indicating that a good coverage of the transcriptome has been achieved. To evaluate the assembly consistency, the filtered unique reads were mapped back to the final assembled leaf transcriptome and the average read mapping rate using the alignment software Bowtie2 was 71.85%. (Table 1). Both transcript and Unigene length distribution is reported in Additional file 2: Figure S3. These data showed that the throughput and sequencing quality were high enough to warrant further analysis.

**Table 1:** Summary statistics of the RNA quality and sequencing results

Average RIN	8
Clean reads	643 million
N° of transcripts	255,809
N° of Unigenes	186,740
Average of read mapped rate	71.85%
Transcripts N50 (bp)	1857
Unigenes N50 (bp)	1845
Q30 (%)	94.7
GC content (%)	53,3

To achieve comprehensive gene functional annotation, all assembled unigenes were blasted against public databases, including National Center for Biotechnology Information (NCBI), Protein family (Pfam), Clusters of Orthologous Groups of proteins (KOG/COG), Swiss-Prot, Ortholog database (KO) and Gene Ontology (GO) (Table 2). A total of 116,488 unigenes were annotated in at least one searched database, accounting for 62.38% of the obtained total unigenes. Among them, 35,630 (19.08%) and 41,101 (22.01%) assembled unigenes showed identity with sequences in the Nr and Nt databases, respectively. The percentage of assembled unigenes homologous to sequences in KO, Swiss-Prot, Pfam, GO and KOG databases were 20.63, 32.41, 26.96, 42.28 and 16.72%, respectively (Table 2).

**Table 2:** The number and percentage of successful annotated genes

Database	Number of Unigenes	Percentage %
Annotated in Nr	35,630	19.08
Annotated in Nt	41,101	22.01
Annotated in KO	38,524	20.63
Annotated in SwissProt	60,522	32.41
Annotated in Pfam	50,345	26.96
Annotated in GO	78,954	42.28
Annotated in KOG	31,223	16.72
Annotated in at least one database	116,488	62.38

### Identification of differentially expressed genes (DEGs)

**Fig. 2:**
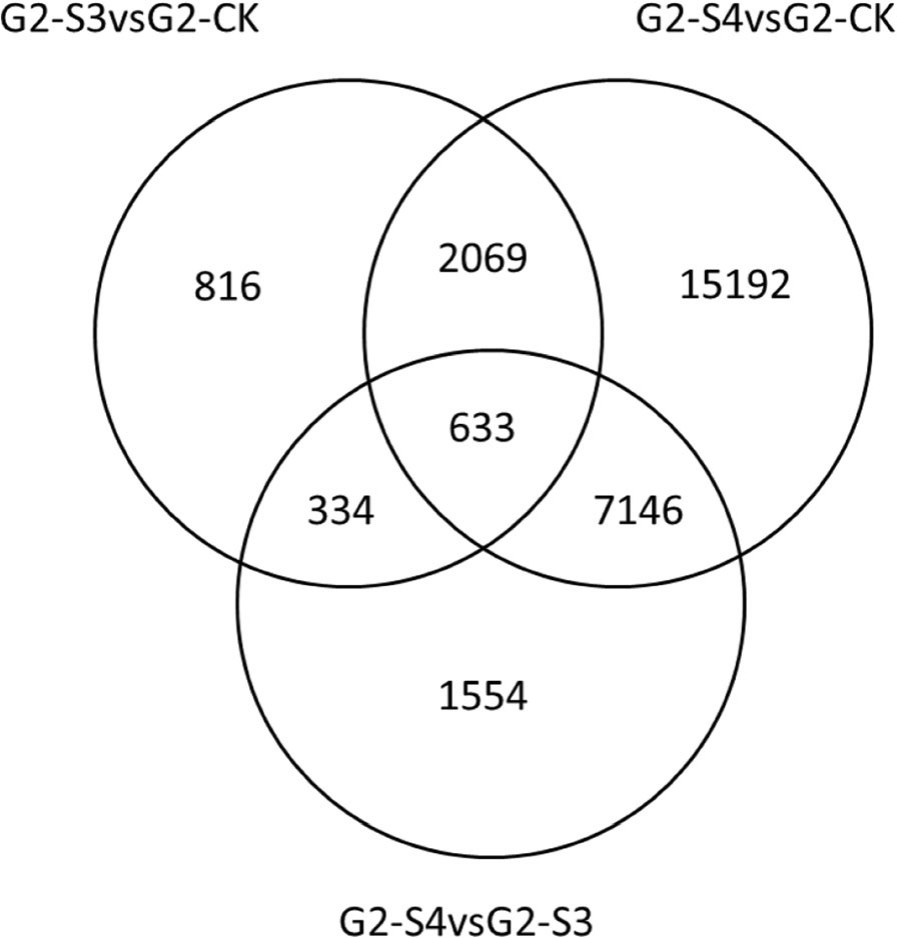
Venn diagram of differently regulated genes. Comparison among G2-S3 vs G2-CK, G2-S4 vs G2-CK, G2-S4 vs G2-S3 sample sets

The characterization of *A. donax* transcriptional response to salt stress was carried out by the identification of the unigenes whose expression level changed upon NaCl treatments (Table 3). According to the experimental design, a total of 38,559 differentially expressed genes (DEGs) were identified from all the comparisons. In details, 2086 up-regulated genes and 1766 down-regulated genes were detected in the G2-S3 vs G2-CK (severe salt stress samples versus control samples), whereas in the G2-S4 vs G2-CK set (extreme salt samples versus control samples) 13,835 up-regulated genes and 11,205 down-regulated genes were found, thus suggesting that G2 clone re-adjusts the network of transcriptional machinery in order to deeply modify gene expression under salt extreme stress conditions (S4) with respect to severe salt conditions (S3) (Table 3). Venn diagram analysis showed that 2702 common DEGs are in both G2-S3 vs G2-CK and in G2-S4 vs G2-CK comparisons thus suggesting that their specific involvement in the response to salt stress is not dependent by salt doses (Fig. 2). A total of 1150 genes are exclusively regulated under severe salt stress condition (G2-S3 vs G2-CK data set), whereas a total of 22,338 genes are specifically regulated during extreme salt stress condition (G2-S4 vs G2-CK data set). Among a total of 9667 observed DEGs, 1554 genes were identified as specifically regulated in the G2-S4 vs G2-S3 (extreme salt samples versus severe salt samples) comparison (Fig. 2). Validation of expression levels for ten selected DEG candidates was carried out by quantitative real-time PCR (qRT-PCR) (Additional file 3: Table S1). The results show high congruence between RNA-Seq results and qRT-PCR (coefficient of determination R^2^ = 0.91) indicating the reliability of RNA-Seq quantification of gene expression (Additional file 3: Figure S4). Therefore, the selected genes could also constitute useful markers of salt stress in *A. donax*.

**Table 3:** DEG number of different comparison under salt treatments

	Up-regulated	Down regulated	Total DEGs
G2-S3 vs G2-CK	2086	1766	3852
G2-S4 vs G2-CK	13,835	11,205	25,040
G2-S4 vs G2-S3	5765	3902	9667
Total DEGs	21,686	16,873	38,559

### Functional classification of DEGs

**Fig. 3:**
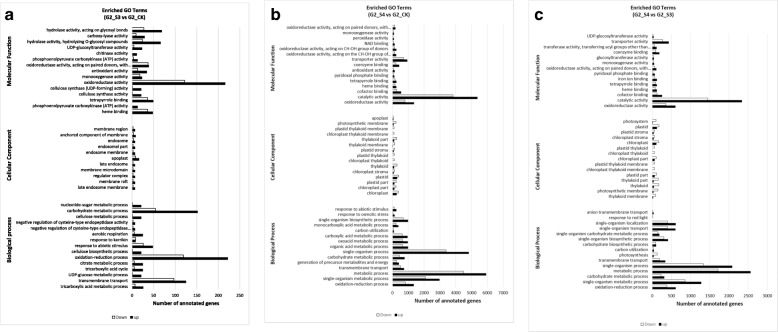
GO enrichment analysis for the DEGs in *A. donax*. **a** G2-S3 vs G2-CK. **b** G2-S4 vs G2-CK. **c** G2-S4 vs G2-S3. The Y-axis indicates the subcategories, and the X-axis indicates the numbers related to the total number of GO terms. BP, biological processes; CC, cellular components; MF, molecular functions

Gene Ontology (GO) terms, Clusters of Orthologous Groups of proteins (KOG) classification and Kyoto Encyclopedia of Genes and Genomes (KEGG) pathway functional enrichment were performed to identify possible biological processes or pathways involved in salt stress response. Considering the G2-S3 vs G2-CK sample set (Fig. 3a), “oxidation-reduction process” (222 up- and 119 down-regulated genes), “transmembrane transport” (125 up- and 97 down-regulated genes) and “carbohydrate metabolic process” (152 up- and 54 down-regulated genes) are the three most enriched GO terms in the Biological Process (BP) ontology. “Oxidoreductase activity” (216 up- and 122 down-regulated genes) is the most enriched GO terms in the Molecular Function (MF) category ontology indicating that genes acting in this process may play crucial roles in the response to salt treatment (Fig. 3a). Among the DEGs belonging to G2-S4 vs G2-CK data set, “metabolic process” (5934 up- and 4493 down-regulated genes), “single organism process” (4834 up- and 3404 down-regulated genes), “single organism metabolic process” (2988 up- and 2090 down-regulated genes) and “oxidation-reduction process” (1355 up- and 831 down-regulated genes) are the most represented in the category of BP. “Catalytic activity” is the main category in the MF group (5380 up- and 3836 down-regulated genes) but also “oxidoreductase activity” and “transporter activity” are highly represented in this group (Fig. 3b). Interestingly, the same categories are represented in the G2-S4 vs G2-S3 sample set (Fig. 3c), although in this last comparison a lower numbers of genes are involved (Fig. 3c). To predict and classify possible functions, all unigenes (255,809) were aligned to the KOG database and were assigned to the KOG categories (Additional file 8: Figure S5). Among the KOG categories, the cluster for “general function” (16%) represented the largest group, followed by “posttranslational modification, protein turnover, chaperones” (13.4%) and “signal transduction mechanisms” (9.3%). “Translation, ribosomal structure and biogenesis” (8%), “RNA processing and modification” (6.4%) and “transcription” (5.6%) were the largest next categories, whereas, only a few unigenes were assigned to “nuclear structure” and “extracellular structure”. In addition, a discrete number of unigenes were assigned to “intracellular trafficking, secretion, and vesicular transport” (Additional file 8: Figure S5). The sets of DEGs originated from the above-described three comparisons were also mapped onto KEGG pathways. Additional file 4: Table S2 shows the main 50 KEGG pathway terms sorted by a decreasing order of the gene number involved in the pathways in relation to all the comparison under investigation (G2-S3 vs G2-CK, G2-S4 vs G2-CK, G2-S4 vs G2-S3). Overall, the results show that the maximum number of DEGs were observed in the “carbon metabolism” pathway, followed by the “biosynthesis of amino acids” and “carbon fixation in photosynthetic organisms” indicating that a deep reprogramming of these metabolisms under salt treatments occurred. The reprogramming activity of the metabolic pathways is supported by the involvement of other important pathways such as “ribosome”, “RNA transport”, “mRNA surveillance pathway” and “aminoacyl-tRNA biosynthesis” that gives support to an increased re-modulation of protein biosynthesis. “Plant hormone signal transduction”, which comprises the transcripts of several hormone-responsive proteins involved in regulation and signal transduction and two other important pathways, including ‘phenylalanine metabolism’ and ‘plant-pathogen interaction’ were also found to be regulated by salt in our study (Additional file 4: Table S2). Other examples of relevant pathways, which are known to be involved in responses to abiotic stresses, in general are ‘starch and sucrose metabolism’, ‘arginine and proline metabolism’ and “AMPK signaling pathway” (Additional file 4: Table S2) [33].

### Identification of functional genes related to salt stress tolerance

To unravel the *A. donax* G2 salt stress response and to investigate the effect of salt dose, we analyzed the RNA-Seq datasets from each of the aforementioned comparisons, focusing on genes and pathways known to be related to soil salinity. In this further analysis, those clusters showing a threshold of +/− 10.000 log_2_fold change have been considered as DEGs (up- or down-regulated) in the *A. donax* transcriptome (Tables 4, 5 and 6). For each cluster, the alignment of *A. donax* sequence has been performed and the score of these alignments was reported (% identity and *e* value) thus providing valuable indications of the cluster similarity with the reported genes (Tables 4, 5 and 6). Congruously, tables report clusters whose % of identity was higher than 50 and the *e* value< 0.05.

**Table 4:** List of DEGs related to salt stress response identified in G2-S3 vs G2-CK comparison

Cluster ID	Database description	Percent identity	Evalue	log_2_ fold change
Salt sensory and signaling mechanisms				
14027.182899	*Oryza sativa subsp. japonica* Group CBL-interacting protein kinase 1 (CIPK1-SOS2 like), (XM_015766590.1)	93.33%	4e-23	+ 39.581
14027.54233	*Setaria italica* probable cation transporter HKT9 (XM_004967183.2)	86.32%	1e-132	+ 18.352
14027.155903	*Phragmites australis* pcnhx1 mRNA for putative Na+/H+ antiporter (NHX1), (Nt ID: AB211145.1)	93.40%	3e-54	− 21.136
14027.181583	*Arabidopsis thaliana* Sodium/hydrogen exchanger 2 (NHX2), (Swissprot ID: Q56XP4)	77.78%	8e-79	− 19.411
Transcription factors				
14027.152638	*Setaria italica* protein TIFY 10B-like (Nr ID: XP_004958300.1)	84.77%	2e-131	− 25.354
14027.159286	*Setaria italica* trihelix transcription factor GTL1 (Nt ID: XM_012842882.1)	88.89%	2e-101	+ 14.963
14027.107730	*Panicum miliaceum* ABSCISIC ACID-INSENSITIVE 5-like protein 7 (Nr ID: RLM78209.1)	78.41%	4e-93	+ 15.107
Hormone regulation of salt stress response				
14027.74861	*Triticum urartu* Abscisic acid 8′-hydroxylase 1 (Nr ID: EMS68885.1)	85.54%	4e-112	+ 47.347
14027.144393	*Setaria italica* abscisic acid receptor PYL8 (Nt ID: XM_004951229.2).	77.85%	4e-80	+ 11.825
14027.137264	*Setaria italica* abscisic acid receptor PYR1-like (Nr ID: XP_004983564.1)	85.34%	1e-104	+ 12.624
14027.173535	*Oryza sativa* serine/threonine-protein kinase SnRK (SnRK), (Swissprot ID: Q75H77)	79.69%	8e-32	+ 14.238
14027.235757	*Setaria italica* probable protein phosphatase 2C 30 (Nt ID: XM_004984868.2)	87.80%	6e-170	+ 27.333
14027.228612	*Setaria italica* cytochrome P450 734A1 (Nr ID: XP_004962129.1)	89.90%	2e-58	+ 16.547
14027.99729	*Saccharum arundinaceum* 1-aminocyclopropane-1-carboxylate oxidase (ACC oxidase) (Nr ID: ABM74187.1)	91.46%	1e-122	+ 15.804
14027.265476	*Setaria italica* indole-3-pyruvate monooxygenase YUCCA2-like (Nr ID: XP_004967465.1)	75.33%	1e-107	− 40.837
ROS scavenging regulatory mechanisms				
14027.179813	*Setaria italica* L-ascorbate peroxidase (Nt ID: XM_004984762.3)	92.70%	2e-67	+ 17.074
14027.36507	*Zea mays* glutathione transferase 23 (GST) (Swissprot ID: Q9FQA3)	86.94%	1e-142	+ 14.004
14027.206288	*Setaria italica* NADP-dependent malic enzyme, chloroplastic (Nr ID: XP_004960887.1)	99.50%	4e-152	+ 15.008
14027.147540	*Setaria italica* malate dehydrogenase, mitochondrial (MDH), (Nt ID: XM_004961089.3)	84.14%	2e-167	+ 12.416
Osmolyte biosynthesis				
14027.146844	*Setaria italica* delta-1-pyrroline-5-carboxylate synthase (P5CS), (Nt ID: XM_004961829.3)	91.92%	6e-168	+ 40.924
14027.163839	*Setaria italica* proline dehydrogenase (PDH) (Nt ID: XM_004983669.2)	89.55%	3e-114	− 28.271
14027.196396	*Oryza sativa Japonica Group* Arginine decarboxylase 1 (ADC) (Nr ID: XP_015643038.1)	92.73%	1e-142	+ 46.553
Photosynthesis and photorespiration				
14027.153819	*Pisum sativum* RuBisCO large subunit-binding protein subunit alpha, chloroplastic (Swissprot ID: P08926)	74.89%	4e-110	− 35.119
14027.193818	*Setaria italica* peroxisomal (S)-2-hydroxy-acid oxidase (Nt ID: XM_004958250.2)	92.51%	5e-156	+ 22.634
Biomass digestibility and biofuel production				
14027.274380	*Setaria italica* cinnamoyl-CoA reductase 2-like (Nt ID: XP_004956337.1)	67.31%	3e-149	+ 39.522
14027.178971	*Zea mays* caffeoyl CoA 3-O-methyltransferase (Nr ID: AAP33129.1)	91.10%	6e-163	+ 13.644
14027.116333	*Setaria italica* sucrose synthase (Nr ID: XP_004984440.1)	70.19%	2e-136	+ 11.104
14027.238308	*Setaria italica* triacylglycerol lipase SDP1-like (Nr ID: XP_004970049.1)	78.83%	6e-112	+ 10.393
14027.166055	*Setaria italica* diacylglycerol kinase 1 (Nr ID: XP_004983987.1)	86.87%	2e-146	− 15.699

**Table 5:** List of DEGs *related to salt stress response identified in G2-S4* vs *G2-CK comparison*

Cluster ID	Database description	Identity score	Identity E value	log_2_ fold change
Salt sensory and signaling mechanisms				
14027.85357	*Setaria italica* CBL-interacting protein kinase 1 (CIPK1-SOS2-like) (Nt ID: XM_004967556.3)	92.31%	8e-73	Inf^a^
14027.198243	*Oryza sativa subsp. japonica* CBL-interacting protein kinase 24 (*SOS2*) (Swissprot ID: Q69Q47)	87.88%	8e-126	+ 11.098
14027.54233	*Setaria italica* probable cation transporter HKT9 (Nr ID: XP_004967240.1)	86.32%	1e-132	+ 35.667
14027.155893	*Arabidopsis thaliana* Sodium/hydrogen exchanger 1 (NHX1) (Swissprot ID: Q68KI4)	68.46%	6e-177	+ 14.324
Transcription factors				
14027.159287	*Setaria italica* trihelix transcription factor GTL1, (Nt ID: XM_012842882.1)	81.72%	6e-45	+ 29.044
14027.82197	*Setaria italica* bZIP ABSCISIC ACID-INSENSITIVE 5-like protein 7 (Swissprot ID: Q9M7Q2)	79.96%	9e-82	+ 17.106
Hormone regulation of salt stress response				
14027.234361	*Setaria italica* abscisic acid 8′-hydroxylase 3 (Nr ID: XP_004957014.1)	83.43%	0.0	+ 41.107
14027.144393	*Setaria italica* abscisic acid receptor PYL8 (Nt ID: XM_004951229.2)	77.85%	4e-80	+ 12.599
14027.36786	*Setaria italica* brassinosteroid-6-oxidase 2 cytochrome P450 85A1 (KO ID: K12640)	93.36%	4e-141	+ 19.415
14027.99735	*Oryza brachyantha* 1-amino cyclopropane-1-carboxylate oxidase (ACC oxidase) (Nt ID: XM_006647913.2)	72.38%	2e-149	+ 22.325
14027.89434	*Oryza sativa Japonica Group* ethylene receptor-like protein 1 (ETR1) (Nr ID: AAL29304.2)	51.05%	3e-48	− 18.141
14027.182197	*Arabidopsis thaliana* serine/threonine-protein kinase CTR1, (Swissprot ID: Q05609)	87.50%	3e-145	+ 11.918
14027.226694	*Setaria italica* ETHYLENE INSENSITIVE 3-like 3 protein (EIN3) (Nr ID: XP_004973869.1)	61.43%	1e-113	+ 14.849
14027.190058	*Zea mays* Indole-3-acetaldehyde oxidase (AAO) (Swissprot ID: O23887)	67.55%	4e-175	+ 13.436
14027.58358	*Setaria italica* probable indole-3-acetic acid-amido synthetase GH3.8 (Nr ID: XP_004958192.1)	69.87%	2e-133	Inf^a^
14027.189947	*Oryza sativa subsp. japonica* Jasmonic acid-amido synthetase JAR1 (Swissprot ID: Q6I581)	60.65%	2e-167	− 28.213
ROS scavenging regulatory mechanisms				
14027.42850	*Flaveria pringlei* NADP-dependent malic enzyme, chloroplastic (Swissprot ID: P36444)	55.29%	6e-111	Inf^a^
14027.184286	*Setaria italica* ubiquinol oxidase 2, mitochondrial-like (AOX) (Nr ID: XP_004976683.1)	93.02%	1e-64	+ 20.525
14027.154629	*Setaria italica* superoxide dismutase [Cu-Zn] (Nt ID: XM_004958551.3)	89.43%	2e-74	+ 36.275
14027.237926	*Setaria italica* superoxide dismutase [Fe] 2, chloroplastic-like (Nt ID: XM_004964461.2)	98.26%	2e-68	− 11.661
Osmolyte biosynthesis				
14027.146844	*Setaria italica* delta-1-pyrroline-5-carboxylate synthase (P5CS) (Nt ID: XM_004961829.3)	91.92%	6e-168	+ 68.438
14027.197137	*Brachypodium distachyon* delta-1-pyrroline-5-carboxylate dehydrogenase 12A1, mitochondrial (P5CSDH) (Nr ID: XP_010231104.1)	92.46%	5e-138	+ 17.285
14027.163909	*Oryza sativa subsp. indica* Betaine aldehyde dehydrogenase (BADH) (Swissprot ID: B3VMC0)	92.96%	1e-41	+ 15.021
5159.0	*Zea mays* ornithine decarboxylase-like (ODC) (Nr ID: XP_008653000.1)	52.96%	1e-112	+ 51.866
14027.196396	*Oryza sativa Japonica Group* Arginine decarboxylase 1 (ADC) (Nr ID: XP_015643038.1)	92.73%	1e-142	+ 65.072
14027.211822	*Setaria italica* spermine synthase-like (SPMS) (Nr ID: XP_004951294.1)	89.78%	2e-175	+ 10.003
Photosynthesis and photorespiration				
14027.158740	*Oryza sativa, subsp. japonica* Group Ribulose bisphosphate carboxylase small chain A (Swissprot ID: P18566)	88.96%	3e-100	− 24.091
14027.70509	*Arundo donax* ribulose-bisphosphate carboxylase large subunit (rbcL) (Nt ID: KJ880079.1)	100.00%	1e-104	− 17.789
14027.158959	*Setaria italica* ruBisCO large subunit-binding protein subunit beta (Nr ID: XP_004975721.1)	95.45%	2e-19	− 14.938
14027.168331	*Setaria italica* ribulose bisphosphate carboxylase/oxygenase activase (Nt ID: XM_004960085.2)	84.71%	1e-46	− 15.647
14027.198015	*Oryza brachyantha* phosphoenolpyruvate carboxylase (PEPC) (Nr ID: XP_006644735)	93,85%	1e-174	+ 11.496
14027.153211	*Oryza sativa* chloroplastic pyruvate phosphate dikinase 1 (PPDK1) (Swissprot ID: Q6AVA8)	88.41%	1e-163	+ 29.711
14027.158029	*Setaria italica* phosphoenolpyruvate carboxylase kinase (PEPC kinase) (Nr ID: XP_004976235.1)	84.05%	2e-127	− 40.627
Biomass digestibility and biofuel production				
14027.150588	*Setaria italica* putative cinnamyl alcohol dehydrogenase (Nt ID: XM_004972526.2)	79.53%	6e-116	+ 14.463

^a^it means that the read count value of CK samples is zero

**Table 6:** List of DEGs *related to salt stress response identified in G2-S4* vs *G2-S3 comparison*

Cluster	Database description	Identity score	Identity Evalue	log_2_ fold change
Salt sensory and signaling mechanisms				
14027.81324	*Setaria italica* probable cation transporter HKT9 (Nr ID: XP_004967240.1)	65.10%	5e-142	+ 25.926
Transcription factors				
14027.184776	*Setaria italica* trihelix transcription factor GTL1 (Nt ID: XM_012842882.1)	80.41%	2e-69	+ 29.515
Hormone regulation of salt stress response				
14027.238560	*Zea mays* 9-cis-epoxycarotenoid dioxygenase 1, chloroplastic (Swissprot ID: O24592)	85.48%	6e-177	− 18.703
14027.234358	*Setaria italica* abscisic acid 8′-hydroxylase 3 (Nr ID: XP_004957014.1)	70.33%	4e-163	+ 23.595
14027.36785	*Setaria italica* brassinosteroid-6-oxidase 2 cytochrome P450 85A1 (KO ID: K12640)	93.12%	2e-105	+ 24.019
ROS scavenging regulatory mechanisms				
14027.104252	*Flaveria pringlei* NADP-dependent malic enzyme, chloroplastic (Swissprot ID: P36444)	70.49%	5e-135	Inf^a^
14027.141579	*Setaria italica* ubiquinol oxidase 2, mitochondrial-like (AOX) (Nr ID: XP_004953576.1)	80.65%	4e-87	+ 13.511
14027.159513	*Zea Mays* superoxide dismutase [Cu-Zn] (Swissprot ID: P23345)	89.67%	2e-164	+ 11.777
14027.172733	*Setaria italica* superoxide dismutase [Fe] 1, chloroplastic-like (Nt ID: XM_004981985.2)	72.96%	1e-165	− 11.799
Osmolyte biosynthesis				
14027.166473	*Setaria italica* delta-1-pyrroline-5-carboxylate synthase (P5CS) (Nt ID: XM_004970516.2)	95.94%	8e-175	+ 44.106
14027.197137	*Brachypodium distachyon* delta-1-pyrroline-5-carboxylate dehydrogenase 12A1, mitochondrial (P5CSDH) (Nr ID: XP_010231104.1)	92.46%	5e-138	+ 17.324
14027.116788	*Oryza sativa* subsp*. japonica* Betaine aldehyde dehydrogenase 1 (BADH) (Swissprot ID: O24174)	85.80%	2e-94	+ 16.655
14027.196396	*Oryza sativa Japonica Group* Arginine decarboxylase 1 (ADC) *(Nr ID: XP_015643038.1)*	92.73%	1e-142	+ 18.455
Photosynthesis and photorespiration				
14027.157747	*Oryza sativa* subsp. j*aponica* Group Ribulose bisphosphate carboxylase small chain A (Swissprot ID: Q0INY7)	94.59%	3e-20	− 13.548
14027.70510	*Adiantum capillus-veneris* ribulose-bisphosphate carboxylase large subunit (rbcL) (Swissprot ID: P36476)	94.33%	9e-180	− 13.342
14027.168328	*Setaria italica* ribulose bisphosphate carboxylase/oxygenase activase (Nt ID: XM_004960085.2)	80.30%	4e-66	− 10.404
14027.153211	*Oryza sativa* subsp. *japonica* chloroplastic pyruvate phosphate dikinase 1 (PPDK1) (Swissprot ID: Q6AVA8)	88.41%	1e-163	+ 28.312
14027.170986	*Setaria italica* phosphoenolpyruvate carboxylase kinase (PEPC kinase) (Nt ID: XM_004953094.3)	83.33%	7e-05	− 17.751
Biomass digestibility and biofuel production				
14027.76193	*Setaria italica* cinnamyl alcohol dehydrogenase (Nt ID: XM_004951572.2)	82.35%	2e-132	− 11.287
14027.238308	*Setaria italica* triacylglycerol lipase SDP1-like (Nr ID: XP_004970049.1)	75.07%	4e-168	+ 10.139

^a^it means that the read count value of CK samples is zero

### Salt sensory and signaling mechanisms

The analysis of different expressed genes between G2-S3 and G2-CK revealed that homologous to *Oryza sativa* (CBL-interacting protein kinases 1) CIPK1-SOS2-like protein and homologous to *Setaria italica* HKT9 gene are up-regulated in the salt treated samples, whereas genes homologous to the *Arabidopsis* NHX1 and NHX2 (Na^+^/H^+^ antiporters) are down regulated in response to severe S3 salt stress (Table 4). CIPK1-SOS2-like protein is a serine/threonine protein kinase involved in the activation of plasma membrane Na^+^/H^+^ antiporter (SOS1) which mediates the exclusion of Na^+^ excess out of the cells, whereas HKT9 gene encodes a probable cation transporter. Consequently, the data suggest that the plant response to the S3 salt dose is likely either to increase the activation upon the existing Na^+^/H^+^ antiporter (SOS1) by the CIPK1-SOS2 like activity, or to adjust the K^+^ homeostasis by inducing the expression of HKT9. The down regulation of NHXs indicates that the vacuolar sequestration of Na^+^ excess seems to be impaired in the *A. donax* subjected to severe salt stress condition. Considering the G2-S4 vs G2-CK data set, a distinct response to extreme salt stress has been detected (Table 5). Along with the up regulation of CIPK1-SOS2 like and HKT9, the induction of CBL-interacting protein kinase 24 SOS2 (homologs of *Oryza sativa subsp. japonica* protein) is also specifically up regulated under extreme salt stress conditions (S4). Morevover, the up regulation of NHX1 expression (*Arabidopsis thaliana* sodium/hydrogen exchanger 1) encoding the vacuolar Na^+^/H^+^ antiporter is observed (Table 5).

### Transcription factors

**Fig. 4:**
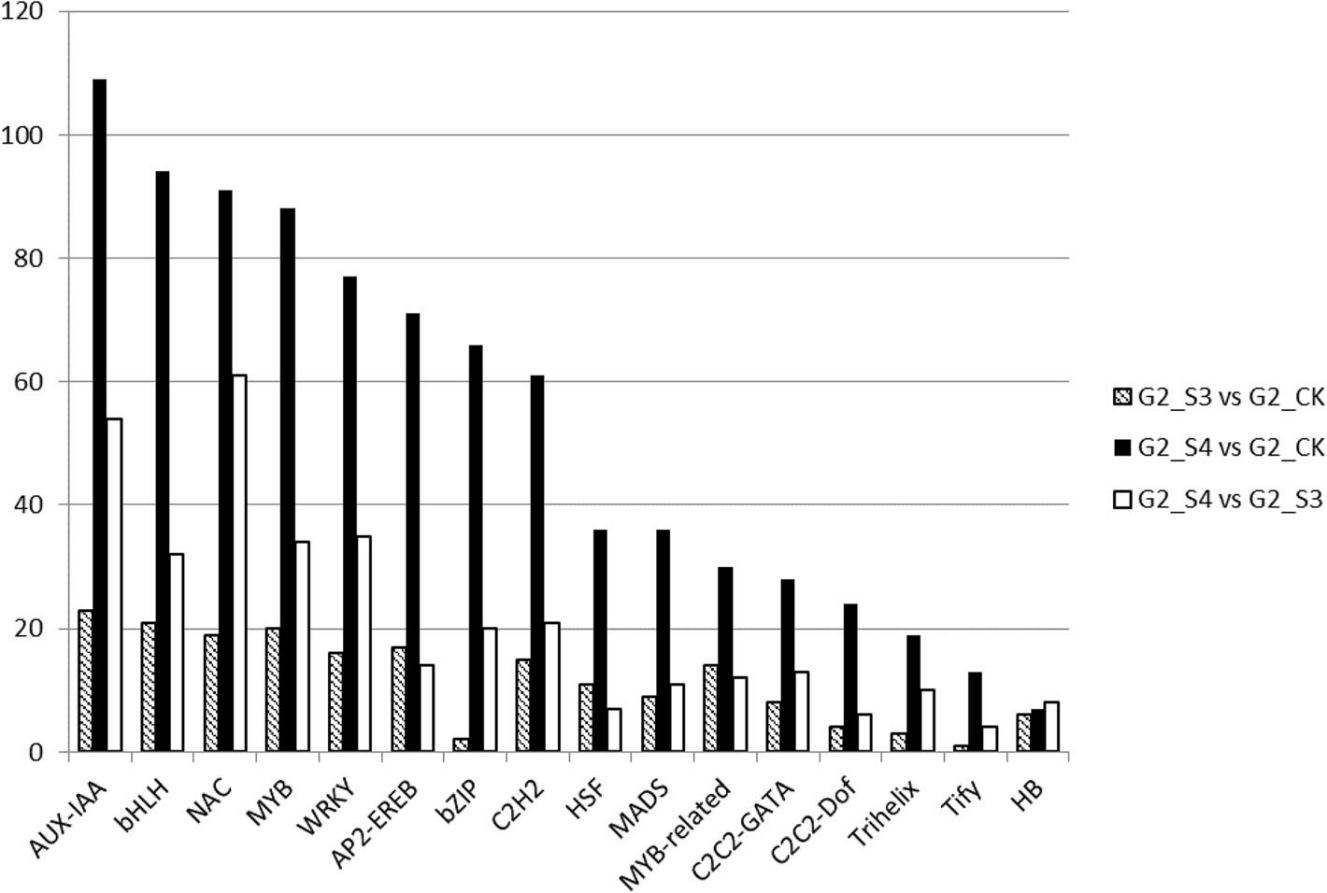
Distribution of transcription factors responsive to salt stress. Data are sorted by number of G2-S4 vs G2 CK DEGs. Only categories with more than 3 DEGs identified as transcription factors are shown

In our study, DEGs encoding TFs were identified and divided in 16 subfamilies, as showed in Fig. 4, which reports the transcription factor subfamilies sorted by the G2-S4 vs G2-CK DEG number. The results showed that under S3 severe stress condition, an average of 12 TFs for each family are differently regulated. In particular, 23 DEGs belong to auxin/indole acetic acid (AUX/IAA), 21 to bHLH and 19 to NAC families, respectively, indicating that these are the most represented subfamilies and they probably play a key role in regulating the changes of transcriptional regulation in response to salt (Fig. 4). It worthwhile to mention that among the downregulated genes belonging to G2-S3 vs G2-CK DEGs, a homolog of *Setaria italica* protein TIFY 10B-like (LOC101761171) known to be a repressor of jasmonate (JA) responses, has been found (Table 4). Moreover, a homolog of *Setaria italica* trihelix transcription factor GTL1 (LOC101762434), that acts as negative regulator of water use efficiency [35, 36] has been found up-regulated in G2-S3 vs G2-CK DEGs (Table 4). Finally, homologs of *Setaria italica* bZIP *ABSCISIC ACID-INSENSITIVE* 5-like protein 7 functioning as transcriptional activator in the ABA-inducible expression of rd29B have been also found among the up-regulated clusters. The role of these differently regulated clusters will be discussed below.

In the G2-S4 vs G2-CK comparison, a deeper modification of the transcription regulation is detected since an average of 53 TFs for each family resulted differently regulated in comparison with untreated samples, being the bHLH (94 DEGs), AUX/IAA (109 DEGs), MYB (88 DEGs) and NAC (91 DEGs) subfamilies the most represented (Fig. 4). A comparative analysis performed using the available database of rice transcription factors under salt stress led to the identification of 449 *A. donax* unigenes, corresponding to high confidence rice TF homologs previously identified as salt or salt/drought genes (Additional file 5: Table S3) [37]. Probably because of the altered water potential under salt stress, the majority of these genes (434) are also responsive to drought. A total of 15 genes are specifically responding to salt (Additional file 5: Table S3), and among them, 9 (six up regulated and three downregulated) belong to the AP2-EREBP family [38]. Interestingly, 14 out of the 15 specific salt-related transcription factors are differently regulated exclusively in extreme salt stress condition (G2-S4 vs G2-CK) (Additional file 5: Table S3).

### Hormone regulation of salt stress response

We focused our attention on the main plant hormones involved in salt stress response, such as abscisic acid, brassinosteroid, ethylene, auxin/IAA and jasmonic acid [39, 40]. The analysis of G2-S3 vs G2-CK data set revealed that the genes involved in abscisic acid biosynthesis (NCED, zeathaxin oxidase and aldehyde oxidase) are not differently regulated by the long term salt stress, otherwise homolog of *Triticum urartu* (Nr ID: EMS68885.1) abscisic acid 8′-hydroxylase 1 (LOC101782596), involved in ABA catabolism, has been found among the up-regulated genes (Table 4). Based on these differential expressions, it seems that circulating ABA is channeled in a degradation pathway and the plant responds to prolonged severe stress by lowering ABA levels. Among the up-regulated genes, homologs of *Setaria italica* abscisic acid receptor PYL8 (LOC101768693), of *Setaria italica* ABSCISIC ACID-INSENSITIVE 5-like protein 7 (LOC101778442), *Setaria italica* abscisic acid receptor PYR1-like (LOC101776342), *Oryza sativa* serine/threonine-protein kinase SnRK (SnRK) and to *Setaria italica* probable protein phosphatase 2C 30 (LOC101766228) were discovered. According to these results, although ABA levels seems to be lowered, as the up regulation of abscisic acid 8′-hydroxylase homolog suggests, ABA signal is probably persisting since ABA nucleoplasmatic receptors are up-regulated as well as the SnRK2 which active many downstream ABA-responsive processes [39]. As regards G2-S4 vs G2-CK and G2-S4 vs G2-S3 sample data, *Setaria italica* abscisic acid 8′-hydroxylase 3 (LOC101760218) and *Setaria italica* abscisic acid receptor PYL8 (LOC101768693) are up-regulated (Tables 5 and 6). Moreover, the downregulation of homolog to *Oryza sativa* 9-cis-epoxycarotenoid dioxygenase (NCED) in the G2-S4 vs G2-S3 comparison, which is not detected in the G2-S3 vs G2-CK, is observed and it might more clearly indicate that ABA synthesis is not induced in samples subjected to long-term extreme salt stress. As regards brassinosteroid, in the G2-S3 vs G2-CK data set, homolog of *Setaria italica* cytochrome P450 734A6-like BAS1 (LOC101760518), a brassinosteroid inactivator, is up-regulated indicating that brassinosteroid signaling is likely interrupted in response to S3 severe salt treatment (Table 4). Conversely, by the analysis of the G2-S4 vs G2-CK data set (Table 5), confirmed by the G2-S4 vs G2-S3 comparison (Table 6), we discovered that homolog of *Setaria italica* cytochrome P450 85A1 (LOC101770408) encoding brassinosteroid-6-oxidase 2, that is implicated in brassinosteroid biosynthesis, is up- regulated under extreme salt stress (Table 5). Transcripts encoding ACC oxidase, namely the ethylene-forming enzyme, have been found up-regulated both under severe (homolog of *Saccharum arundinaceum* 1-aminocyclopropane-1-carboxylate oxidase) and extreme salt stress (homolog of *Oryza brachyantha* 1-aminocyclopropane-1-carboxylate oxidase (LOC102702913). However, in G2-S3 vs G2-CK sample data, none of the ethylene downstream acting genes have been found, neither among the up-regulated nor among the down-regulated clusters (Table 4). Conversely, the analysis of the G2-S4 vs G2-CK revealed that homolog of *Arabidopsis thaliana* receptor ethylene response 1 (ETR 1) is downregulated, whereas clusters related to CTR1 (*Zea mays* serine/threonine-protein kinase CTR1-like) and EIN3 (*Setaria italica* ETHYLENE INSENSITIVE 3-like 3 protein), both implicated in quenching the ethylene signal, are up-regulated (Table 5). Sharp differences between the response of *A. donax* to salt dose have been detected once we considered the role of auxin/IAA as signal molecule. In this respect, the genes encoding the main biosynthetic enzyme, such as indole-3-pyruvate monooxygenase YUCCA2-like (LOC101757189) [41], is downregulated in G2-S3 vs G2-CK comparison indicating that severe salt stress seems not to implicate an increase in IAA levels. Analyzing the response to extreme salt stress upon *A. donax* leaves, we found a strong induction of different clusters relative to YUCCA-like indole-3-pyruvate monooxygenases, which have been described as a high redundant gene family [41]. Unfortunely, several clusters are also found among the downregulated genes thus rendering difficult to make general conclusions (data not shown). However, a homolog of *Zea mays* indole-3-acetaldehyde oxidase (AAO), involved in the biosynthesis of auxin, is upregulated in S4 samples and also in the G2-S4 vs G2-S3 comparison suggesting that IAA might be synthesized in S4 extreme conditions (Table 6). Moreover, exclusively under extreme stress condition a homolog of auxin responsive GH3 gene family, regulating levels of biologically active auxin, is also up-regulated (Table 5). Finally, homolog of *Oryza sativa subsp. japonica* jasmonic acid-amido synthetase JAR1 is down regulated in G2-S4 vs G2-CK comparison (Table 5) but not in the G2-S3 vs G2-CK samples indicating that under extreme salt stress conditions jasmonic acid biosynthesis might be impaired.

### ROS scavenging regulatory mechanisms

The analysis of G2-S3 vs G2-CK revealed that, among the antioxidant enzymes, ascorbate peroxidase (APX) expression is up-regulated suggesting that H_2_O_2_ could be the main ROS the *A. donax* cells have to cope with under severe salt stress (Table 4). Many GSTs are also up-regulated concordantly with their role in salt stress relief [42, 43]. Also the plastid NADP-malic dehydrogenase, reducing oxalacetate to malate, thus regenerating the NADP^+^, is among the up-regulated genes of the G2-S3 vs G2-CK data set, this result being consistent with the electron drainage from an over-reduced photosynthetic chain to other cellular compartments, in particular towards the mitochondria. Indeed, homolog of the mitochondrial malate dehydrogenase (MHD) are also induced by salinity (Table 4). As concerns either the G2-S4 vs G2-CK or G2-S4 vs G2-S3 comparisons (Tables 5 and 6), the plastid NADP-malic dehydrogenase and alternative oxidase (AOX), that transfers electrons towards the respiratory electron chain for energy dissipation, are unequivocally up regulated under extreme salt stress conditions.

### Osmolyte biosynthesis

The accumulation of compatible osmolytes, such as proline, glycine betaine, polyamines and sugar alcohols plays a key role in maintaining the low intracellular osmotic potential of plants and in preventing the harmful effects of salinity stress [23, 40, 44]. In our study, 1-delta-pyrroline-5-carboxylate synthase (P5CS), the key enzyme of proline biosynthesis, was found up regulated in all comparisons (G2-S3 vs G2-CK, G2-S4 vs G2-CK and G2-S4 vs G2-S3) (Tables 4, 5 and 6) suggesting that proline accumulation might represent a pivotal mechanism to overcome the hypersaline conditions and adjust the osmotic status in *A. donax*. Consistent with its catabolic role, proline dehydrogenase (PDH) expression is down regulated in G2-S3 vs G2-CK data set suggesting that the mitochondrial degradation of proline is prevented (Table 4). Conversely, PDH and 1-delta-pyrroline-5-carboxylate dehydrogenase (P5CSDH), both involved in proline catabolism, resulted up regulated in both G2-S4 vs G2-CK and G2-S4 vs G2-S3 data sets (Tables 5 and 6). Biosynthesis pathway of betaine comprises a two steps oxidation of choline in which betaine aldehyde dehydrogenase (BADH) synthesizes betaine from betaine aldehyde. BADH expression is up regulated in G2-S4 vs G2-CK and G2-S4 vs G2-S3 comparisons indicating that betaine might play a crucial role under S4 extreme stress conditions (Tables 5 and 6). Due to their cationic nature, polyamines can interact with proteins, nucleic acids, membrane phospholipids and cell wall constituents, either activating or stabilizing these molecules. Considering all the comparison data set, clusters encoding arginine decarboxylase (ADC, polyamine biosynthetic enzyme) are up-regulated suggesting that polyamines biosynthesis is induced during long term salt stress in *A.donax*, having most likely a role in the salt tolerance mechanism, both under severe and extreme stress condition (Tables 4, 5 and 6). In addition, clusters homolog to ornithine decarboxylase (ODC) and to spermine synthase (SPMS) are exclusively up-regulated in G2-S4 vs G2-CK comparison, indicating that a stronger activation of the polyamine biosynthesis occurs under extreme salt stress, also involving the polyamine biosynthetic pathway starting from ornithine by the action of ODC (Table 5). Sugar alcohols are compatible solutes classified into two major types cyclic (e.g., pinitol) and acyclic (e.g., mannitol) [40]. In our study, neither mannitol-1-phosphate dehydrogenase (*mldh*) nor inositol methyl transferase (*imt*) transcripts, respectively involved in mannitol and pinitol biosynthesis, were in the all the comparisons under investigation, indicating that the synthesis of these compounds might be not crucial for salt stress overcoming in *A. donax*.

### Photosynthesis and photorespiration

The analysis of G2-S3 vs G2-CK DEGs reveals that homologs of both small and large subunits of ribulose-1,5-bisphoshate carboxylase/oxygenase (Rubisco) are not represented neither in the up-regulated or in the down regulated clusters (Table 4). However, homologs of *Pisum sativum* Rubisco large subunit-binding protein subunit alpha, required for the correct assembly of Rubisco, have been discovered among the down-regulated genes, indicating that the process of Rubisco assembly could be strongly affected under S3 salt stress (Table 4). Moreover, clusters encoding glycolate oxidase (*Setaria italica* peroxisomal (S)-2-hydroxy-acid oxidase, LOC101764130), which is a key enzyme of the glycolate recovery pathway induced by photorespiration, is up-regulated in G2-S3 vs G2-CK comparison suggesting that CO_2_ assimilation via the C3 Calvin cycle might be impaired in favor of oxygen fixation through the photorespiration pathway (Table 4). This result is consistent with the decrease of net photosynthesis showed in Additional file 1: Figure S1. Conversely, a surprising scenario takes place in the case of *A. donax* samples subjected to extreme salt stress conditions (Table 5). Mainly, clusters encoding both the small and the large Rubisco subunits (homologs of *Oryza sativa* ribulose bisphosphate carboxylase small chain), clusters encoding *Arundo donax* Rubisco large subunit-binding proteins (involved in Rubisco assembly) and *Setaria italica* ribulose bisphosphate carboxylase/oxygenase activase, are among the down-regulated genes. Rubisco activase plays an important role adjusting the conformation of the active center of Rubisco by removing tightly bound inhibitors thus contributing to the enzyme rapid carboxylation [45]. These findings indicate that extreme salt treatment induce a strong slowdown if not even a dramatic stop of the C3 Calvin cycle (Tables 5 and 6). However, specifically under S4 extreme salt stress, among the up-regulated DEGs, homologs of *Flaveria trinervia* phosphoenolpyruvate carboxylase (PEPC) and of *Oryza sativa* chloroplastic pyruvate phosphate dikinase 1 (PPDK1) have been identified, both involved in C4 photosynthesis, in which the spatial separation of the initial fixation of atmospheric CO_2_ from the Calvin cycle occurs. Concordantly, homologs of *Setaria italica* phosphoenolpyruvate carboxylase kinase 2-like (LOC101779241), the PEPC inactivating enzyme by decreasing of maximal reaction rate, were found down-regulated, suggesting that giant reed response to extreme salt stress tends to maximize the catalytic efficiency of PEPC (Tables 5 and 6).

### Biomass digestibility and biofuel production

Considering the economical relevance that bioenergy crops assume as source of bioethanol, we analyzed the regulation of several genes involved in the improvement of lignocellulosic biomass. In the *A. donax* transcriptome subjected to both severe and extreme salt stress, several homologs of phenylpropanoid biosynthetic genes were highly expressed (Table 4, 5 and 6). In particular, homologs of *Setaria italica* cinnamoyl-CoA reductase 2-like and *Zea mays* caffeoyl CoA 3-O-methyltransferase are among the up-regulated clusters in G2-S3 vs G2-CK samples, being both specifically involved in lignin biosynthesis (Tables 5 and 6) [46]. Similarly, homolog of *Setaria italica* cinnamyl alcohol dehydrogenase was observed among the up-regulated clusters in the G2-S4 vs G2- CK but also in G2-S4 vs G2-S3 sample indicating that lignin biosynthesis is induced under extreme salt stress condition. Besides the homologs of the phenylpropanoid pathway discussed above, we identified transcripts homologous to sucrose synthase (*Setaria italica* sucrose synthase) in the G2-S3 vs G2-CK comparison, a key enzyme in cellulose biosynthesis. Considered that also lipids can participate to biomass yield, we focused our attention on genes encoding key enzymes such as triacylglycerol lipase and diacylglycerol kinase that were found to be up regulated under severe salt stress (Table 4).

### Retrieval and analysis of genes targeted as “salt stress responsive”

The GO terms were further analyzed in order to retrieve clusters specifically involved in the salt stress response, thus excluding all transcripts also regulated by different abiotic stress, such as water deprivation, cold, heavy metals and oxidative stresses (geneontology.org/). All the retrieved clusters are also found to be involved in salt-induced osmotic stress according to the finding that levels of NaCl higher than 100–150 mM cause osmotic stress, that normally arises up at salinity levels ranging between 50 and 100 mM NaCl [47]. The results shown in Additional file 6: Table S4 reveal that among 9 clusters specifically regulated by salt, 7 are up regulated and 2 are down regulated in the G2-S3 vs G2-CK comparison. Among the up regulated genes, the CBL-interacting protein kinase 1 (CIPK1-SOS2 like) has been found (Additional file 6: Table S4) thus suggesting that it induces specific signal transduction pathways under severe salt stress conditions. Instead, clusters related to plasma membrane Na^+^/K^+^ transporter are down regulated by severe salt stress treatment (Additional file 6: Table S4), both results being already highlighted in Table 4. A higher number of up and down regulated genes have been found among the G2-S4 vs G2-CK data set, they being 29 and 7, respectively, for a total of 36 clusters (Additional file 6: Table S4). Similarly to G2-S3 vs G2-CK comparison, clusters encoding the CBL-interacting protein kinase 1 (CIPK1-SOS2 like) have been found up regulated under extreme salt stress S4 (Additional file 6: Table S4, Table 5). Moreover, CBL-interacting protein kinase 24 SOS2 (homolog of *Oryza sativa subsp. japonica* protein, Table 5) is specifically up regulated under extreme salt stress conditions (S4). Interestingly, clusters encoding homologs of the mitochondrial persulfide dioxygenase ETHE1 (ETHYLMALONIC ENCEPHALOPATHY PROTEIN1), that catalyzes the oxidation of persulfides derived from either cysteine or hydrogen sulfide to thiosulfate and sulfate [48] have been found up regulated in S4 conditions. Finally, clusters mainly related to a probable *Oryza sativa subsp. japonica* cation transporter HKT6 are down regulated under extreme salt conditions (Additional file 6: Table S4). In order to support the relationship among the main specific salt responsive genes (CIPK1-SOS2 like, cation transporter HKT9, NHX1, NHX2, SOS2 and ETHE 1, Table 4 and Additional file 6: Table S4) and their orthologues, each *A.donax* cluster was aligned with 15 orthologues from different plant sources and phylogenetic trees were constructed (Additional file 9: Figure S6). The sequence alignments allowed to classify the proteins within the respective protein family and, in the case of CIPK1-SOS2 like, of persulfide dioxygenase ETHE1 and CBL-interacting protein kinase 24 (SOS2) sequence alignment revealed the presence of specific protein functional domains (Additional file 7: Table S5). The findings of the phylogenetic trees depicted that all the genes from different plant sources can be subcategorized into subgroups (Additional file 9: Figure S6a-f) and that, all the giant reed genes were clustered into one of these subgroups.

## Discussion

Plants generate high yields if the growth demands are properly supplied as well as light and temperature fit to their optimum requirements. Yield-associated traits are inversely related to abiotic stress conditions such as salt during plant development. Under conditions of moderate salinity (EC 4–8 dS m^− 1^), all important glycophytic crops reduce average yields by 50–80% [49]. Plants have developed the ability to sense both the hyperosmotic component and the toxic ionic Na^+^ component of salt stress [23]. To date the molecular identities of plant hyperosmotic sensors and Na^+^ sensors present at the plasma membrane have remained unknown. Recently, Choi et al. [50] suggested that Ca^2+^-dependent signaling plays a role in the systemic transmission of signaling as a Ca^2+^ wave propagates preferentially through cortical and endodermal cells from roots to distal shoots. In salt tolerant plants, the cytosolic calcium perturbation actives the Salt Overly Sensitive (SOS) pathway [51, 52]. The components of this pathway are the Ca^2+^ sensor (SOS3) which accordingly changes its conformation in a Ca^2+^-dependent manner and interacts with SOS2, a serine/threonine protein kinase, forming the active SOS2-SOS3 complex. This interaction results in the activation through its phosphorylation of SOS1 (plasma membrane Na^+^/H^+^ antiporter) which mediates the exclusion of Na^+^ excess out of the cells. In addition, the SOS2-SOS3 complex activates NHX, the vacuolar Na^+^/H^+^ exchanger resulting in the vacuolar sequestration of Na^+^ excess thus further contributing to the restore of ion homeostasis [53, 54]. Consequently, the data suggest that the SOS pathway is only partially activated under severe salt stress (up-regulation of CIPK1-SOS2-like protein and HKT9) and the down regulation of NHX indicates that the vacuolar sequestration of Na^+^ excess seems to be impaired (Table 4). A specific response to extreme salt stress has been detected (Table 5), since the up regulation of CIPK1-SOS2 like and HKT9 (*Setaria italica* probable cation transporter HKT9) was accompanied by the up-regulation of NHX1 (*Arabidopsis thaliana* sodium/hydrogen exchanger 1) encoding the vacuolar Na^+^/H^+^ antiporter. Moreover, CBL-interacting protein kinase 24 (homologs of *Oryza sativa subsp. japonica* protein, Table 5), involved in the regulatory pathway for the control of intracellular Na^+^ and K^+^ homeostasis and salt tolerance, is specifically up regulated under extreme salt stress conditions (S4). It activates the vacuolar H^+^/Ca^2+^ antiporter and operates in synergy with CBL4/SOS3 to activate the plasma membrane Na^+^/H+ antiporter SOS1 [55]. As expected, the components of the SOS response are among the salt induced specific genes, indicating their key role in salt detoxification. In addition to that, several transcripts homologous to *Arabidopsis* NHX5 and NHX6 encoding endosomal Na^+^/H^+^ antiporters are also up-regulated (data not shown). Although the relative log_2_ fold changes of these clusters are below the + 10.000 threshold we established at the beginning of the analysis, these results anyway suggest that the cellular components devoted to Na^+^ excess expulsion, located in the tonoplast and in the endosomal membranes all together might participate in reducing the Na^+^ cytoplasmic concentrations. The importance of these genes in salt stress relief is supported by the finding that *Arabidopsis nhx5 nhx6* double knockout showed reduced growth and increased sensitivity to salinity [56]. Downstream of aforementioned activation of Ca^2+^ alteration induced by salinity, kinases become activated and may transduce the hyperosmotic signal to induce protein activities and gene transcription. The activation of transcription factors can occur by the direct binding with calmodulin-binding transcriptional activators (CAMTAs), GT-element binding proteins and MYBs [23, 57]. Transcription factors (TFs) are considered as the most important regulators controlling the expression of a broad range of target genes ultimately influencing the level of salt tolerance in plants. It is well documented that TFs belonging to the DREB, NAC, MYB, MYC, C2H2 zinc finger, bZIP, AP2/ERF (Ethylene Responsive Factor) and WRKY families are relevant in salt stress response [39]. In most cases, the overexpression of these transcription factors successfully enhanced salinity tolerance in many crops [24]. By comparing our results with those obtained in *A. donax* subjected to water deficit [33], slight differences can be observed in terms of TF subfamilies involved in salt and water stresses, but a greater number of all TFs for each family resulted differently regulated under both severe and extreme salt stress. Interestingly, a major involvement of AUX/IAA TFs is detected under salt stress with respect to *A. donax* plants subjected to drought thus indicating that a different regulation network is induced. Moreover, a total of 449 *A. donax* unigenes correspond to high confidence rice homologs previously identified as salt or salt/drought responsive genes (Additional file 5: Table S3) [37]. Most of them (434) resulted also responsive to drought, indicating that the plant responses to these stresses probably overlap each other and that the downstream metabolic pathways can crosstalk. Significantly, few genes (15) specifically respond to salt treatments and they have been found especially (14 out of 15) among the G2-S4 vs G2-CK DEGs (Additional file 5: Table S3), suggesting they might have a crucial role in the response to extreme salt conditions. Among these clusters, 9 (six up regulated and three downregulated) belong to the AP2-EREBP family [38]. They have been implicated in various hormones-related signal transduction pathway including abscisic acid (ABA), ethylene and jasmonates (JAs) [38], which seem to be strongly involved in *A. donax* extreme salt stress response.

In our study, among the downregulated genes belonging to G2-S3 vs G2-CK DEGs, a homolog of *Setaria italica* protein TIFY 10B-like (LOC101761171) known to be a repressor of jasmonate (JA) responses, has been found (Table 4). As detailed below, an important attribute of JA is its ability to act both as a potent inhibitor of vegetative growth and as a positive regulator of reproductive and defensive processes [58]. These antagonistic JA activities suggest that, in *A. donax* subjected to severe salt stress, the dilemma of plant “to grow or defend itself” seems to be resolved trying to growth in an unfavorable environment. A homolog of *Setaria italica* trihelix transcription factor GTL1 (LOC101762434) that acts as negative regulator of water use efficiency via the promotion of stomatal density and distribution [35, 36] has been found up-regulated in G2-S3 vs G2-CK DEGs (Table 4). It has been reported that GTL1 is expressed when *Arabidopsis* plants have sufficient available water but is downregulated by water deficit. *Arabidopsis thaliana* GTL1 loss-of-function mutations result in increased water deficit tolerance and higher integrated water use efficiency by reducing daytime transpiration without a demonstrable reduction in biomass accumulation. Moreover, GTL1 does not regulate ABA responsiveness, and the lower transpiration rates of *gtl1* defective plants are not caused by differences in stomatal aperture or ABA-induced stomatal closure [35]. The observed up-regulation of GTL1 under S3 severe salt stress reveals the susceptibility of *A. donax* plants to salt-induced water deficit and that it might be a target for gene knockout in order to genetically improve the *A. donax* performance in salty soil. Finally, homologs of *Setaria italica* bZIP *ABSCISIC ACID-INSENSITIVE* 5-like protein 7 functioning as transcriptional activator in the ABA-inducible expression of rd29B have been also found among the up-regulated clusters. Although their role is still unknown, rd29B proteins has potential to confer abiotic stress resistance in crop species grown in arid and semi-arid regions [59].

**Fig. 5:**
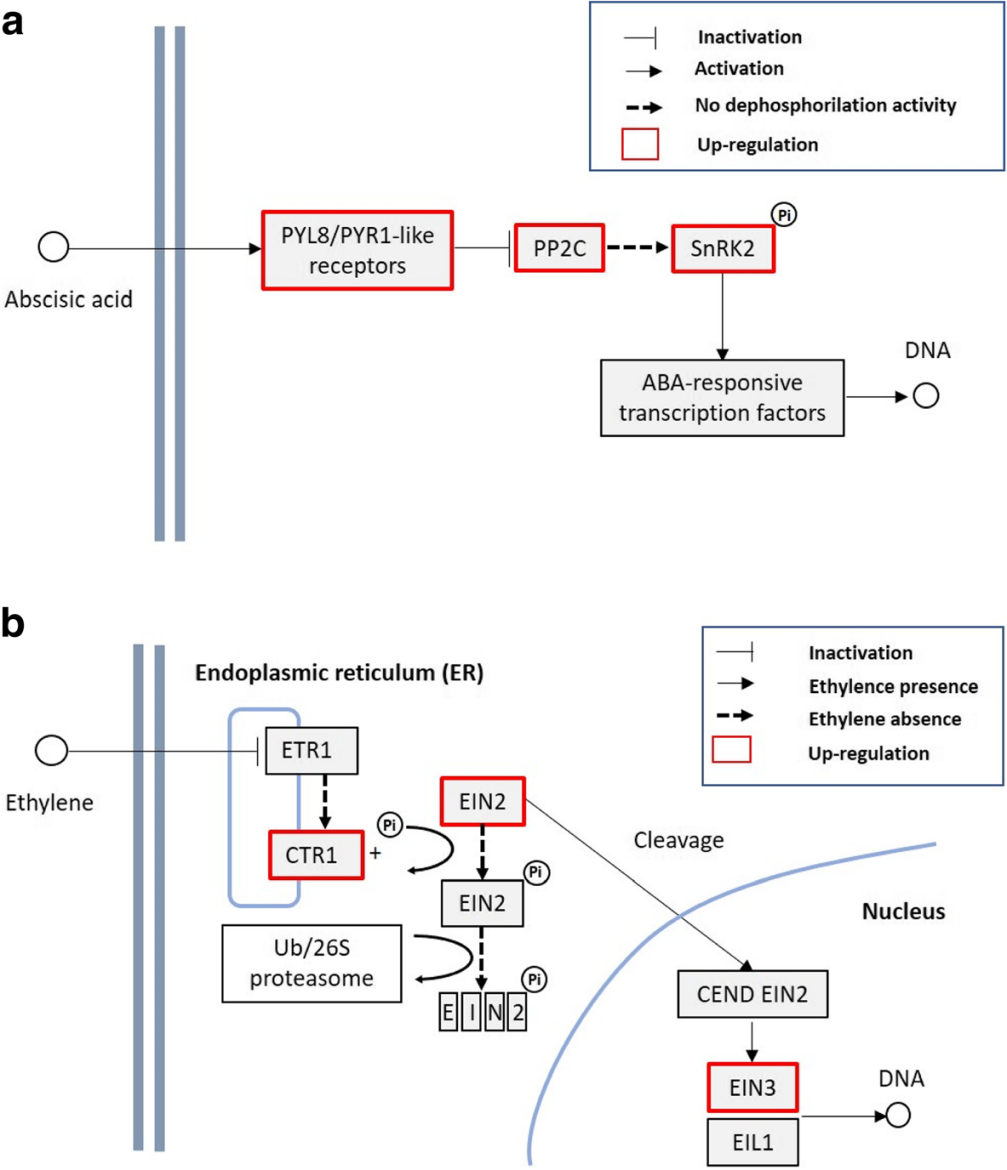
Abscisic acid and ethylene signal pathways. **a** abscisic acid receptor (PYL8), abscisic acid receptor (PYR1-like), serine/threonine-protein kinase (SnRK); protein phosphatase 2C (PP2C). The PYR/PYL/RCARs receptors bind ABA and inhibit type 2C protein phosphatases (PP2C). The active form of SnRK2 accumulates and positively regulates ABA-responsive metabolic pathways. **b** ethylene response 1 (ETR1), kinase constitutive triple response 1 protein (CTR1), putative metal transporter ethylene insensitive 2 (EIN2), EIN2 C-terminal fragment (CEND EIN2), ethylene insensitive 3 (EIN3) and EIN3 like proteins (EIL1). In absence of ethylene, CTR1 directly interacts with ETR1 and phosphorylates EIN2 that is in turn degraded. In the presence of ethylene, CTR1 is inactivated and the EIN2 dephosphorylated form is proteolytically cleaved to generate the CEND EIN2, initiating transcriptional regulation involving ethylene insensitive 3 (EIN3) and EIN3 like proteins (EIL1). See the text for details

The synthesis, sequestration, transportation, and turnover of hormones generate a net of signals that correlates plant growth in dependence on internal and external cues. Among these hormones, abscisic acid regulates important abiotic stress responses, in particular water balance and osmotic stress tolerance under drought and salt stress [60]. ABA levels depend on the equilibrium between synthesis and degradation pathways. Several ABA biosynthetic genes have been isolated from different sources including zeathanxin epoxidase (ABA1), 9-cis-epoxycarotenoid dioxygenase (NCED), ABA aldehyde oxidase (AOX) and ABA3/LOS5 [61]. The hydroxylation at the 8′-position of ABA is known as the key step of ABA catabolism, and this reaction is catalyzed by ABA 8′-hydroxylase, a cytochrome P450 [62]. ABA signals are perceived by different cellular receptors operating in distinct cellular compartments (Fig. 5a). The PYR/PYL/RCARs receptors bind ABA and inhibit type 2C protein phosphatases (PP2C) [39]. The PP2C inactivation leads to the accumulation of the active form of SNF1-RELATED PROTEIN KINASE (SnRK2) which in turn positively regulates ABA-responsive transcription factor as well the downstream ABA-responsive metabolic pathways. In this context, the analysis of G2-S3 vs G2-CK means to propose that plant responds to prolonged severe stress by lowering ABA levels (Table 4). However, ABA signal is probably persisting since ABA nucleoplasmatic receptors are up-regulated as well as the PP2C and SnRK2 which, as detailed before, active many downstream ABA-responsive processes [39] (Fig. 5a). Recently, the increase of leaf tissue ABA concentrations at two h after plants were exposed to 50 mM of the ions was observed in maize indicating that ABA synthesis and accumulation are part of the early response to salt [63]. Interestingly, the putative ortholog of AT1G78390, nine-cis-epoxycarotenoid dioxygenase 9 (NCED), is strongly upregulated in both *Arundo* shoots and roots during the early responses to water stress [33], this finding being in line with our suggestion that ABA might be not strictly involved in the response to a prolonged salt stress treatment. Brassinosteroids are growth-promoting plant hormones that act to enhance cell expansion and increase tolerance to stresses, including salinity, by mediating the synthesis of enzymatic or non-enzymatic antioxidant systems, proline, or lectins [64]. The specific induction under extreme salt stress (S4) but not in severe (S3) conditions of cytochrome P450 85A1 (LOC101770408) encoding brassinosteroid-6-oxidase 2, involved in active brassinosteroid biosynthesis might suggest a key role of these hormones in the case extreme environmental conditions are reached. Ethylene is biosynthesized by the plant in response to life-cycle events or environmental cues including among other diseases, mechanical stress, drought or flood. The phenotypes that can be observed with respect to ethylene signaling typically relate to the inhibition of plant growth and seasonal changes in a plant’s life cycle. Ethylene is efficiently biosynthesized from 1-aminocyclopropane-1-carboxylic acid (ACC) [65]. The mechanism of ethylene action, from perception to function, has been referred to as the “cleave and shuttle model” [66] (Fig. 5b). The most studied of the receptors is ethylene response 1 (ETR1) and downstream of ETR1 *in A. thaliana* is the kinase constitutive triple response 1 protein (CTR1) [67]. In absence of ethylene, this protein directly interacts with the ethylene receptors and it is required to be localized to the endoplasmic reticulum and be kinase active to be signaling active. CTR1 phosphorylates the putative metal transporter ethylene insensitive 2 (EIN2) that triggers its degradation by the Ub/26S proteasome [67]. In the presence of ethylene, CTR1 is inactivated by the interaction with ETR1 and the EIN2 dephosphorylated form is proteolytically cleaved to generate a C-terminal fragment called CEND EIN2. The CEND fragment localizes to the nucleus and initiates transcriptional regulation involving ethylene insensitive 3 (EIN3) and EIN3 like proteins (EIL1) (Fig. 5b). Interestingly, transcripts encoding ACC oxidase have been found up-regulated both under severe and extreme salt stress, whereas homolog of *Arabidopsis thaliana* ETR is downregulated and clusters related to CTR1 and EIN3 are exclusively up-regulated in G2-S4 vs G2-CK samples (Table 5). In our opinion, this condition might describe a situation in which a low perception of emitted ethylene is attempted under extreme salt stress (down regulation of ETR1 expression), concomitantly to an increased expression of CTR1, of EIN2 and EIN3 with the aim to minimize the negative effect of ethylene upon plant growth. Salt also alters the expression of auxin responsive genes and auxin/IAA pathways in different plants [68] conferring higher tolerance to NaCl treatment [69]. Auxin is assumed to activate the proton pump of the plasma membrane pumping protons from the cytosol into the apoplast, resulting in wall loosening and an increase in wall extensibility [69]. In this respect, a homolog of *Zea mays* indole-3-acetaldehyde oxidase (AAO), involved in the biosynthesis of auxin, is upregulated in S4 samples and also in the G2-S4 vs G2-S3 comparison suggesting that IAA is synthesized in S4 extreme conditions (Table 6). Moreover, exclusively under extreme stress condition (Table 5) a homolog of auxin responsive GH3 gene family is also up-regulated. All the results, considering the role of GH3 genes in regulating levels of biologically active auxin through amino acid conjugation, thereby targeting them for degradation [70], indicate that auxin level might be finely tuned under S4 extreme stress condition. Globally, the high number of AUX/IAA transcription factors differently regulated in extreme salt environment accounts for a key role of auxins during long-term salt treatment (Fig. 4). Finally, jasmonic acid-amido synthetase JAR1 is exclusively down regulated under extreme salt stress (Table 5), indicating that jasmonic acid signaling is likely impaired. The relationship between the salt stress response and the JA pathway is not well understood at molecular and cellular levels. However, large-scale transcriptomic studies have shown JA signaling pathway is activated by salt stress leading to root growth inhibition in *Arabidopsis* [71]. The observed down regulation of JAR1 (Table 5) indicates that the pathway involved in the inhibition of root elongation might be not activated in *A. donax,* likely to address the plant demand to explore soil in the attempt of avoiding stress.

A consequence of high salt in the soil is the generation of a low water potential zone around the roots area making extremely difficult for the plants to obtain water and nutrients. Stomatal closure occurs in order to low water loss by transpiration, but it is at the same time responsible of sharp decrease in CO_2_ availability for Calvin cycle and a depletion of oxidized NADP^+^. The overproduced electrons are transferred to O_2_ to generate O_2_^●-^ and a series of dangerous oxygen reactive species (ROSs) causing unrestricted oxidation of various cellular components such as membrane lipids, proteins, and nucleic acid [72]. Therefore, salinity tolerance is positively correlated with the induction of ROS scavenging enzymes such as superoxide dismutase (SOD), catalase (CAT), glutathione peroxidase (GPX), ascorbate peroxidase (APX), monodehydroascorbate reductase (MDHAR), dehydroascorbate reductase (DHAR) and glutathione transferases (GSTs) [40]. Moreover, under stress conditions, the activation of malate-oxalacetate (OAA) shuttle permits the transfer of reducing equivalents among compartments. In particular, the plastid NADP-malate dehydrogenase reduces oxalacetate to malate, thus regenerating the NADP^+^. Malate is then translocated into the cytoplasm where is converted back in oxaloacetate, generating NADH by the cytosolic malate dehydrogenase. This called malate-valve appears to play a primary role under salinity constituting an important mechanism in salt acclimation [73]. Drainage of electron flow towards the AOX oxidase pathway increases under salt stress [74] and prevents over-reduction of ubiquinone thus lowering excessive ROS generations. The malate-valve seems to be activated under S3 severe salt stress conditions (up-regulation of plastid NADP-malic dehydrogenase encoding gene), this result being consistent with a putative electron drainage from the over-reduced photosynthetic chain to other cellular compartments, in particular towards the mitochondria as homolog of the mitochondrial malate dehydrogenase (MHD) are also induced by salinity (Table 4). Under extreme salt stress, the unequivocal up regulation of the NADP-malic dehydrogenase and AOX oxidase is probably aimed to move the excess of reducing power from plastids to the cytosol and to avoid over-reduction of the mitochondrial respiratory chain. However, most of the other considered clusters related with the cellular antioxidant machinery have been found between both the down and up-regulated clusters. In our opinion, this is because under extreme stress conditions, which is certainly an emergency condition, a discerning regulation is needed both among and inside the cell compartments. To support our hypothesis, the SOD plastid pool comprising Cu/Zn SOD and Fe/SOD results differently regulated, with Cu/Zn SOD up regulated and Fe/SOD down regulated (Tables 5 and 6). As multifunctional amino acid, proline seems to have diverse roles under stress conditions, such as stabilization of proteins, membranes, and subcellular structures, and protecting cellular functions by scavenging ROSs. Biosynthesis of proline occurs in the chloroplast or cytosol via glutamate pathway in which 1-delta-pyrroline-5-carboxylate synthase (P5CS) catalyzes the key regulatory and rate limiting reaction [75]. During proline synthesis, 2 mol of NADPH per mole are consumed thus draining electrons from chloroplasts and contributing to the stabilization of redox balance and maintenance of cellular homeostasis when electron transport chain is saturated because of adverse conditions. Proline catabolism occurs predominantly in the mitochondria involving proline dehydrogenase (PDH) or proline oxidase (POX) and 1-delta-pyrroline-5-carboxylate dehydrogenase (P5CSDH). The PDH and P5CSDH use NAD and FAD as electron acceptor, respectively, that deliver electrons to the respiratory chain to gain energy and resume growth after stress [75]. The up-regulation of P5CS found in all comparisons suggests that that proline biosynthesis represents a pivotal mechanism to overcome the hypersaline conditions and adjust the osmotic status in *A. donax*. However, since PDH and P5CSDH resulted up regulated in extreme salt stress conditions (Tables 5 and 6), we propose that S4 salt dose triggers a specific response that is not related to the mere proline synthesis to cope with osmotic stress. Regarding this aspect, it has been shown that proline catabolism is enhanced during stress recovery attempts. During this phase, it might function as signaling molecule proposed to regulate the expression of stress recovery genes [76]. Therefore, under extreme salt stress, proline levels might be accurately regulated in order to accommodate the whole cell demand in terms of both osmotic potential and redox homeostasis adjustments. Polyamines play a crucial role in abiotic stress tolerance including salinity and increases in the level of polyamines are correlated with stress tolerance in plants [77, 78]. The comparison of all data sets indicates that polyamines biosynthesis is induced during long term salt stress in *A.donax* having most likely a role in salt tolerance mechanism, especially under extreme stress condition (Tables 4, 5 and 6). Photosynthesis is the primary processes to be affected by salinity [22, 79]. Stomata close in response to leaf turgor declines, therefore supply of CO_2_ to Rubisco (EC 4.1.1.39) is impaired thus inducing sharp alterations of photosynthetic metabolism. In *A. donax* under severe salt stress condition, CO_2_ assimilation via the C3 Calvin cycle seems to be impaired in favor of oxygen fixation through the photorespiration pathway. Moreover, the findings indicate that extreme salt treatment induces a down regulation of all C3 Calvin cycle enzymes and a concomitant switch on of C4 photosynthesis. The induction of PEPC activity and its expression following salt stress is documented in the facultative CAM plant *Mesembryanthemum crystallinum* and it is involved in the change from C3 to CAM photosynthesis [80]. In *A. donax* leaves, the activation of C4 pathway associated to a down-regulation of Rubisco biosynthesis, assembly and activation could be construed as an ultimate rescue attempt to overcome the long term extreme conditions.

As concern metabolic pathways related to bioenergy production, it has been shown that lignin content in cell wall is inversely related to yield and conversion efficiency of polysaccharides into ethanol [46]. Unfortunately, in the *A. donax* transcriptome subjected to both severe and extreme salt stress, several cluster involved in lignin biosynthesis are induced and this unwanted consequence of soil salinization might negatively affect biomass digestibility. This unfavorable circumstance could be compensated by the fact that, after biomass saccharification, lignin residue can be used to produce biodegradable plastic and chemicals [81]. Furthermore, the induction of transcripts homologous to sucrose synthase (*Setaria italica* sucrose synthase) in the G2-S3 vs G2-CK comparison accounts for a probable increase in cellulose content that it has been shown to be without negative effects on growth [82]. Recently, the lipid fraction has been proposed as pivotal component of green biomass since it stores twice as much energy than cellulose per unit of weight [83]. The up-regulation of key enzymes, such as triacylglycerol lipase and diacylglycerol kinase under severe salt stress supports the hypothesis that *A. donax* G2 response is trying to cope stress by inducing gene expression of pathways involved in biomass yield. The further analysis performed to identify genes which are regulated uniquely under salt stress conditions highlighted that a very small subset of clusters are up or down regulated by salt and not by other abiotic stress thus suggesting that the response pathways to different environmental cues often cross-talk and overlap each other in plants. As expected, the main salt-specific pathways are related to the SOS response [51, 52] and to the activation of ETHE1 [84] (Additional file 6: Table S4). In *Arabidopsis* leaves, ETHE1 sulfur dioxygenase has a key function in the degradation of sulfur-containing amino acids and strongly affects the oxidation of branched-chain aminoacids as alternative respiratory substrates in situations of carbohydrate starvation [84]. Therefore, ETHE1 could be relevant for stress tolerance against soil salinity in giant reed.

## Conclusions

The possibility to assign marginal land to bioenergy crop cultivation represents the main strategy to overcome the forthcoming conflict between land demand for feeding the world population and the request of new energy sources to sustain it. Salt affected soils are a widespread agricultural problem limiting crop production due to ionic, osmotic and oxidative stresses with negative impact on plant growth. In this work, the bioenergy crop *A. donax*, known to be able to growth in unfavorable environments, was subjected to two levels of long-term salt stress both doses being much higher than that used to define a soil area as “salinized”. To cultivate bioenergy crops in such soils might represent the unique possibility of their utilization, releasing suitable soil for crop cultivation. Moreover, considering that in S4 extreme salt treatment, the Na^+^ is very close to that of seawater, we propose, as water-saving strategy, to irrigate the soil allocated to *A. donax* cultivation with opportune seawater dilutions. To fill the lack of information about the molecular mechanism involved in *A.donax* response to salt stress, we de novo sequenced, assembled and analyzed the *A. donax* G2 leaf transcriptome in response to the above detailed salt stresses. The response to salt and other environmental constrains such as drought share similar attributes. However, we found that most of the *A. donax* annotated DEGs are homologs of genes belonging also to other species (*Setaria italica* and *Zea mays*) thus suggesting that long term salt stress regulates a specific set of genes providing a general overview of the prolonged salt stress transcriptional responses in *A. donax* (Tables 4, 5 and 6). The picture that emerges from the identification of functional genes related to salt stress is consistent with a dose-dependent response to salt. The number of DEGs under extreme salt stress is much higher than that observed in severe salt stress suggesting that a deep re-programming of the gene expression must occur in S4 samples, which, during the experiment, certainly grew in an “*emergency*” state. As concerns hormone regulation, the response to S3 severe salt stress seems not to be dependent by ABA levels as gene involved in its biosynthesis are not differently regulated whereas the clusters encoding the main catabolic enzyme are strongly up-regulated. Moreover, once *A. donax* plants were subjected to S4 extreme salt stress a clear down regulation of ABA biosynthetic genes is registered suggesting that ABA synthesis might have a key role during the onset of stressful conditions as demonstrated in other species [63] and in the case of water stress [33] but not in the case of long term stress. Another distinct trait of the *A. donax* response to S4 extreme salt stress is the induction of clusters involved in brassinosteroid and IAA/AUX biosynthesis which probably have a key role in those more unfavorable conditions. Similarly, the down-regulation of gene involved in jasmonic acid biosynthesis suggests that JA signaling, leading to root growth inhibition, is repressed likely in the attempt to let the roots explore the surrounding soil more efficiently. The analysis of clusters related to ethylene biosynthesis and signaling indicated that, exclusively under S4 extreme salt stress, the gene transcription is modulated towards the minimization of ethylene negative effects upon plant growth. The *A. donax* leaves subjected to S3 severe salt stress respond to salt-induced oxidative stress by the induction of genes involved in ROS scavenging (APX) and in redistributing the reducing power excess among cell compartments (malate valve). Along with the clusters implicated in the malate valve, under S4 extreme salt stress, also gene encoding the alternative oxidase (AOX) have been found up regulated, highlighting once more that the induction of some pathways occurs in the case of more stringent environmental conditions. A clear involvement of proline and polyamines in coping the salt-induced osmotic stress can be suggested whereas sugars seem not to be involved as osmolytes protecting cell homeostasis. Certainly, the photosynthesis and photorespiration processes are strongly affected since under S3 severe salt treatment, genes involved in Rubisco assembly are down-regulated, and the fact that *A. donax* leaf are impeded to operate CO_2_ fixation via C3 Calvin cycle is also supported by the up-regulation of genes involved in photorespiration (glycolate oxidase). Conversely, in S4 extreme salt treated samples, a dramatic change from C3 Calvin cycle to C4 photosynthesis is likely to occur as all gene regulation is addressed to repress Rubisco synthesis and assembly, and to activate the primary CO_2_ fixation to PEP in mesophyll cells (C4 pathway), this probably being the main finding of our work. Considered the distinct response to salt dose, either genes involved in S3 severe or in S4 extreme salt response could constitute useful markers of the physiological status of *A. donax* in salinized soil. Moreover, many of the unigenes identified in the present study have the potential to be used for the development of novel *A. donax* varieties with improved productivity and stress tolerance, in particular the knock out of the GTL1 gene acting as negative regulator of water use efficiency has been proposed as good target for genome editing experiments.

## Methods

### Plant material and application of salt stress

The experiment was conducted at the Department of Agriculture, Food and Environment (Di3A) of the University of Catania, initially using three different giant reed clones, namely G2, G18 and G20 ecotypes, originated from Caltagirone (Italy), (latitude 37°14′, longitude 14°31′), Biancavilla (Italy) (latitude 37°38′, longitude 14°52′) and Capo D’ Orlando (Italy) (latitude 38°08′, longitude 14°43′), respectively, and collected for the *Giant reed Network* project [14]. The trial started on July 7th, 2017, by transplanting *A. donax* rhizomes into 25 l pots (40 cm diameter and 30 cm height) containing a sandy soil as substrate. Before transplantation, the rhizomes were weighed using a laboratory scale and the number of buds was counted. For each ecotypes, samples showing homogeneous rhizome weight and same bud number were used for transplanting. The individual rhizomes were then placed at 15 cm depth, one for each pot. The pots were arranged according to a randomized block factor scheme, performing three biological replicates for treatments. During the experiment, the irrigation was performed on a weekly basis, and until the first sprouts have been released, tap water (5 l per pot) has been used. Irrigation was carried out manually using a watering can, avoiding possible leaks by leaching. The first irrigation with saline water was carried out on August 3rd, 2017. Salt stress was imposed by adding different concentrations of NaCl to the irrigation water. In particular, S0 samples (no salt added), S3 (severe salt stress, 256.67 mM NaCl corresponding to 32 dS m^− 1^ electric conducibility, EC), S4 (extreme salt stress, 419.23 mM NaCl corresponding to 50 dS m^− 1^ electric conducibility, EC), this last concentration being very close to the seawater NaCl concentration (3%), where Na^+^ molarity is about 460 mM and Cl^−^ is around 540 mM [57]. Before leaf harvest, the following morpho-biometric and physiological parameters were measured: number of culms, height of the main culm, number of green and senescent leaves, net photosynthesis and chlorophyll content measured in SPAD units (SPAD 502, Konica Minolta). Moreover, the measurement of the yielded biomass was also carried out [14]. The collected data were submitted to ANOVA analysis, using CoStat 6.003 software. The averages were separated by the Student Newman Keuls (SNK) test when *P* ≤ 0.05. On the basis of the aforementioned parameters suggesting contrasting behavior under salinity stress (S3 and S4 salt levels) among the clones under investigation, G2 was selected to perform the global transcriptomic analysis.

### Sample collection and RNA extraction

In November 17th, 2017, fully expanded, no senescing G2 leaves (the 3rd leaf from the top) were harvested and immediately frozen with liquid nitrogen. Then, plant material, kept frozen by continuously liquid nitrogen adding, was ground using precooled mortar and pestle followed by RNA isolation using the Spectrum Plant Total RNA Extraction Kit (Sigma-Aldrich, St. Louis, MO, USA) according to the manufacturer’s instructions. RNA degradation and contamination were monitored on 1% agarose gels. RNA purity and concentration were assayed using the NanoDrop spectrophotometer (ThermoFisher Scientific, Waltham, MA, USA). RNA integrity was assessed using the Agilent Bioanalyzer 2100 system (Agilent Technologies, Santa Clara, CA, USA). Before to be sequenced, the RNA samples were subjected to quality parameter evaluation. The average RNA Integrity Number (RIN) was of 8.0 and there was very slight genomic DNA contamination confirming that all the samples have such high quality level to be processed (Table 1).

### Library preparation for transcriptome sequencing

A total amount of 1.5 μg RNA per sample was used as input material for the RNA sample preparations. Sequencing libraries were generated using NEBNext® Ultra™ RNA Library Prep Kit for Illumina® (New England Biolabs, Ipswich, MA, USA) following manufacturer’s recommendations. Briefly, mRNA was purified from total RNA using poly-T oligo-attached magnetic beads. Fragmentation was carried out using divalent cations under elevated temperature in NEBNext First Strand Synthesis Reaction Buffer (5X). First strand cDNA was synthesized using random hexamer primer and M-MuLV Reverse Transcriptase (RNase H-). Second strand cDNA synthesis was subsequently performed using DNA Polymerase I and RNase H. Remaining overhangs were converted into blunt ends via exonuclease/polymerase activities. After adenylation of 3′ ends of DNA fragments, NEBNext Adaptor with hairpin loop structure were ligated to prepare for hybridization. In order to select cDNA fragments of preferentially 150~200 bp in length, the library fragments were purified with AMPure XP system (Beckman Coulter, Beverly, MA, USA). Then 3 μl USER Enzyme by NEB was used with size-selected, adaptor-ligated cDNA at 37 °C for 15 min followed by 5 min at 95 °C before PCR. Then PCR was performed with Phusion High-Fidelity DNA polymerase, Universal PCR primers and Index (X) Primer. At last, PCR products were purified (AMPure XP system) and library quality was assessed on the Agilent Bioanalyzer 2100 system.

### Clustering and next generation RNA sequencing

Cluster generation and sequencing were performed by Novogene Bioinformatics Technology Co., Ltd. (Beijing, China). The clustering of the index-coded samples was performed on a cBot Cluster Generation System using a PE Cluster kit cBot-HS (Illumina). After cluster generation, the library preparations were sequenced on Illumina HiSeq2000 platform to generate pair-end reads. Raw data (raw reads) of fastq format were firstly processed through in-house perl scripts. In this step, clean data were obtained by removing reads containing adapter, reads containing ploy-N and low-quality reads. At the same time, Q20, Q30, GC-content and sequence duplication level of the clean data were calculated. All the downstream analyses were based on clean data with high quality (Fig. 1).

### De novo transcriptome assembling and gene functional annotation

De novo transcriptome assembly was accomplished using Trinity (r20140413p1 version) with min_kmer_cov:5 parameters (k = 25). Then Hierarchical Clustering was performed by Corset (v1.05 version) to remove redundancy (parameter -m 10). Afterwards the longest transcripts of each cluster were selected as Unigenes. Gene function was annotated based on the following databases: National Center for Biotechnology Information (NCBI) non-redundant protein sequences (Nr), NCBI non-redundant nucleotide sequences (Nt), Protein family (Pfam), Clusters of Orthologous Groups of proteins (KOG/COG), Swiss-Prot, Kyoto Encyclopedia of Genes and Genomes (KEGG), Ortholog database (KO) and Gene Ontology (GO) (Fig.1).

### Identification of clusters specifically involved in the salt stress response

In order to discriminate among clusters specifically regulated by salt treatment from those also involved in the response to other abiotic stress (oxidative, water deprivation stress, cold, heavy metals), the GO term lists relative to each comparison (G2_S3 vs G2_CK and G2_S4 vs G2_CK) were filtered and exclusively salt-regulated clusters were extrapolated. For the identification of transcription factors responsive to salt stress in *A. donax*, we mined the available salt stress-responsive transcription factor database of rice (SRTFDB) [35] by Blastn searches with an *e* value cutoff of 1e− 5.

### Multiple sequence alignment and phylogenetic analysis

Multiple alignment analysis of 15 amino acid sequences of selected proteins (CIPK1-SOS2 like, cation transporter HKT9, NHX1, NHX2, SOS2 and ETHE 1) was carried out by MUSCLE by executing MEGA X 10.0.5 (https://www.megasoftware.net/). The phylogenetic tree was created using MEGA X 10.0.5 by the ML (maximum likelihood) method following the Jones, Taylor and Thornton (JTT) substitution model and 1000 bootstrap replicate with other default parameters.

### Quantification of gene expression and differential expression analysis

Gene expression levels were estimated by RSEM (v1.2.26 version) with bowtie2 mismatch 0 parameters in order to map to Corset filtered transcriptome. For each sample, clean data were mapped back onto the assembled transcriptome and readcount for each gene was then obtained from the mapping results. Differential expression analysis between control and salt stressed samples was performed using the DESeq R package (1.12.0 version, padj< 0.05). The resulting *p-*values were adjusted using the Benjamini and Hochberg’s approach for controlling the false discovery rate. Genes with an adjusted *p*-value < 0.05 found by DESeq were assigned as differentially expressed. The GO enrichment analysis of the differentially expressed genes (DEGs) was implemented by the GOseq R packages (1.10.0, 2.10.0 version, corrected *P*-Value< 0.05 based) Wallenius non-central hyper-geometric distribution [85]. Furthermore, to analyze the *Arundo donax* transcriptome all of the unigenes were submitted to the KEGG pathway database for the systematic analysis of gene functions. KOBAS software (v2.0.12 version, corrected P-Value< 0.05) was used to test the statistical enrichment of differential expression genes in KEGG pathways.

### Real-time validation of selected DEG candidates using qRT-PCR

Total RNA (2.5 μg) extracted from sample leaves as described above, was reversed transcribed using the SuperScript™ Vilo™ cDNA synthesis kit by ThermoFisher Scientific, according to the manufacturer’s instructions. Real-time qRT-PCR was performed for a total of 10 DEGs with PowerUp SYBR Green Master mix by ThermoFisher Scientific and carried out in the Bio-Rad iQ5 Thermal Cycler detection system. All the genes were normalized with *A. donax* 26 S proteasome non-ATPase regulatory subunit 11 gene (RPN6) that was reported to be a suitable housekeeping gene in abiotic stress conditions [86]. All reactions were performed in triplicate and fold change measurements calculated with the 2^−ΔΔCT^ method. Sequences of primers used for real-time PCR are provided in Additional file 3: Table S1.

## Additional files

Additional file 1: **Figure S1.** Effect of salt stress upon G2, G18 and G20 ecotype morpho-biometric and physiological parameters. . a Leaf number per pot. b Stem number per pot. c Main stem height. d SPAD. e Net photosynthesis. f Dry biomass (PDF 97 kb)

Additional file 2: **Figure S2.** Picture of giant reed phenotype under salt stress. **Figure S3.** Length distribution of transcripts and Unigenes (ZIP 1076 kb)

Additional file 3: **Table S1. and Figure S4.**. Validation of *A. donax* DEGs by Real Time qRT-PCR (XLSX 14 kb)

Additional file 4: **Table S2.** Distribution of KEGG pathways for DEGs in the three sample sets. (DOCX 15 kb)

Additional file 5: **Table S3.** Transcription factors responsive to salt, to drought or both to salt and drought stresses in *A. donax (XLSX 44 kb)*

Additional file 6: **Table S4.** Clusters specific regulated by salinity (XLSX 23 kb)

Additional file 7: **Table S5.** Protein family and domain description. (XLSX 10 kb)

Additional file 8: **Figure S5.** Kog functional classification. Clusters of orthologous groups (KOG) classification. All unigenes were aligned to KOG database to predict and classify possible functions. Out of 255,809 unigenes, 49,848 sequences were assigned to 25 KOG classifications. (A) RNA processing and modification; (B) chromatin structure and dynamics; (C) energy production and conversion; (D) cell cycle control, cell division, chromosome partitioning; (E) amino acid transport and metabolism; (F) nucleotide transport and metabolism; (G) carbohydrate transport and metabolism; (H) coenzyme transport and metabolism; (I) lipid transport and metabolism; (J) transition, ribosomal structure and biogenesis; (K) transcription; (L) replication, recombination and repair; (M) cell wall/membrane/envelope biogenesis; (N) cell motility; (O) posttranslational modification, protein turnover, chaperones; (P) inorganic ion transport and metabolism; (Q) secondary metabolites biosynthesis, transport and catabolism; (R) general function prediction only; (S) function unknown; (T) signal transduction mechanisms; (U) intracellular trafficking, secretion, and vesicular transport; (V) defense mechanisms; (W) extracellular structures; (X) unnamed protein; (Y) nuclear structure; (Z) cytoskeleton. (RTF 5034 kb)

Additional file 9: **Figure S6.** Phylogenetic relationship among *A. donax* salt responsive clusters and orthologues belonging to different plant sources. **a** cluster 14,027–155,903 homolog of *Phramites australis* Na^+^/H^+^ antiporter (NHX1). **b** cluster 14,027–181,583 homolog of *Arabidopsis thaliana* Na^+^/H^+^ exchanger 2 (NHX2). **c** cluster 14,027–182,899 homolog of *Oryza sativa* CBL-interacting protein kinase 1 (CIPK1-SOS2-like). **d** cluster 14,027–182,899 homolog of *Oryza sativa* CBL-interacting protein kinase 24 (SOS2). **e** cluster 14,027–54,233 homolog of *Setaria italica* cation transporter (HKT9). **f** cluster 14,027–231,386 homolog of *Arabidopsis thaliana* persulfide dioxygenase (ETHE1). (DOCX 171 kb)

## Data Availability

The *Arundo donax* transcriptome was submitted to NCBI (https://www.ncbi.nlm.nih.gov/geo/) accession number GSE121552.

## Abbreviations

ABA: Abscisic acid
AUX/IAA: Auxin/indole acetic acid
BP: Biological process
DEGs: Differentially expressed genes
ERF: Ethylene responsive factor
ETHE1: ETHYLMALONIC ENCEPHALOPATHY PROTEIN1
GHG: Greenhouse gases emission
GO: Gene ontology
GTL1: Trihelix transcription factor GTL1
JA: Jasmonic acid
KEGG: Kyoto encyclopedia of genes and genomes
KO: Ortholog database
KOG/COG: Clusters of orthologous groups of proteins
MF: Molecular function
NCBI: National center for biotechnology information
Nr: NCBI non-redundant protein sequences
Nt: NCBI non-redundant nucleotide sequences
OPTIMA: Optimization of Perennial Grasses for Biomass Production in the Mediterranean Area
PEPC: Phosphoenolpyruvate carboxylase
Pfam: Protein family
qRT-PCR: Quantitative real-time PCR
RIN: RNA integrity number
RNA-seq: RNA sequencing
ROS: Reactive oxygen species
Rubisco: Ribulose-1,5-bisphosphate carboxylase/oxygenase
S0: No salt stress
S3: Severe salt stress
S4: Extreme salt stress
SOS: Salt overly sensitive
TFs: Transcription factors
