# Supplementary figures and images for: RNASeq analysis of giant cane reveals the leaf transcriptome dynamics under long-term salt stress
Source: BMC Plant Biol. 2019 Aug 15;19:355. doi: 10.1186/s12870-019-1964-y (PMC6694640; doi:10.1186/s12870-019-1964-y)

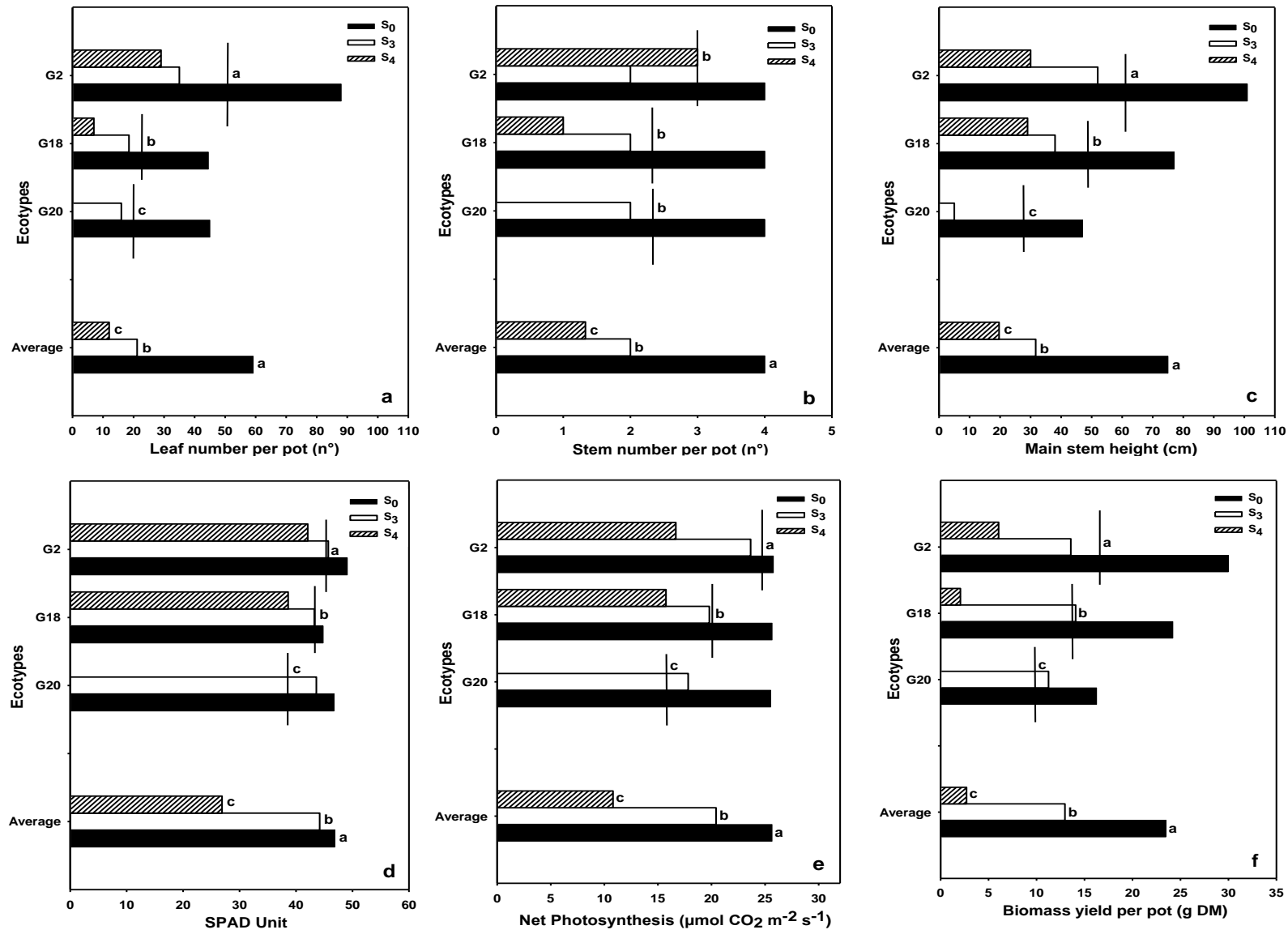

Figure S1

Supplement: Supplementary file 1 — Figure S1. Effect of salt stress upon G2, G18 and G20 ecotype morpho-biometric and physiological parameters. . a Leaf number per pot. b Stem number per pot. c Main stem height. d SPAD. e Net photosynthesis. f Dry biomass (PDF 97 kb) [file 12870_2019_1964_MOESM1_ESM.pdf]

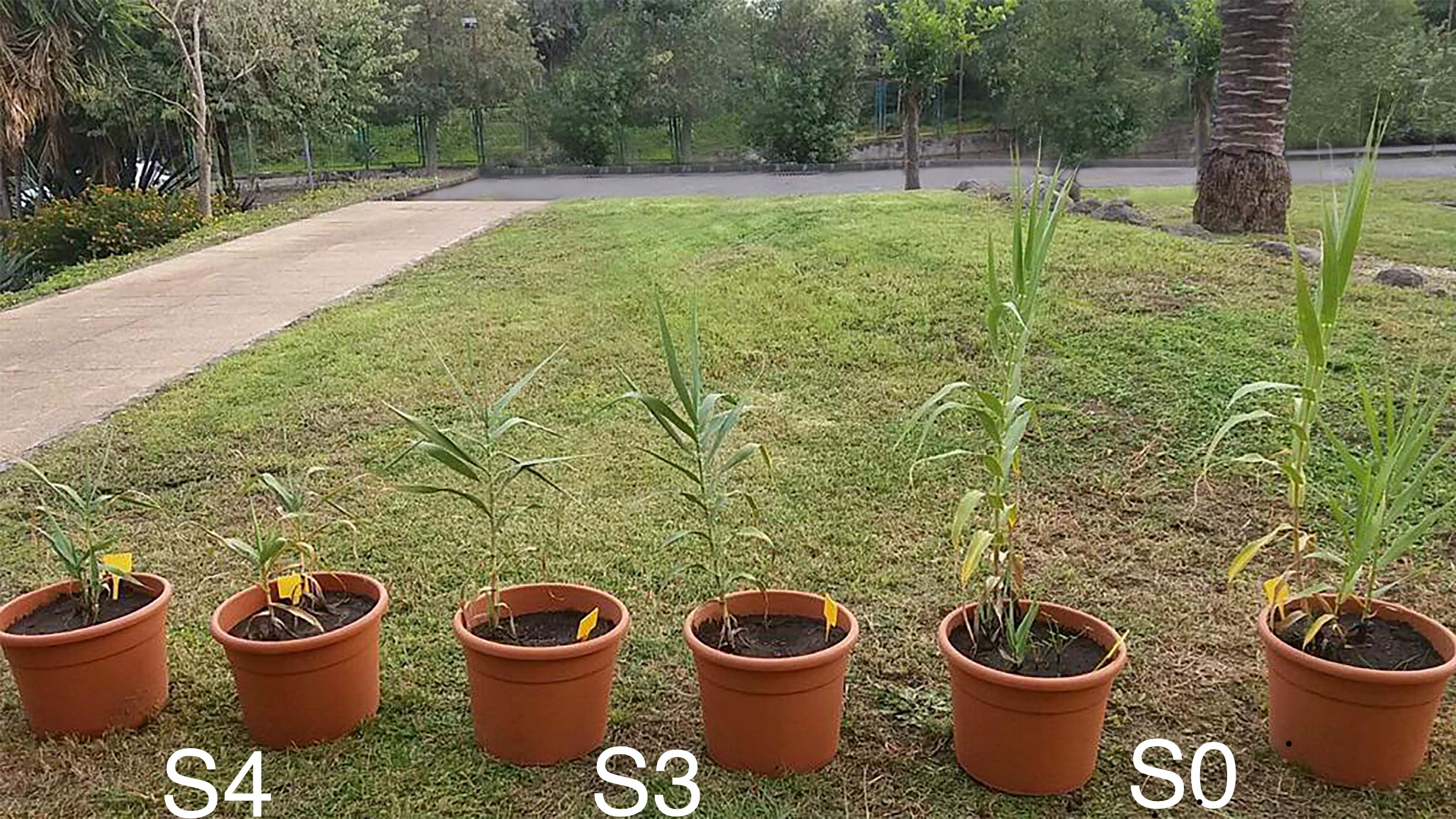

Supplement: Supplementary file 2 — Figure S2. Picture of giant reed phenotype under salt stress. Figure S3. Length distribution of transcripts and Unigenes (ZIP 1076 kb) [file 12870_2019_1964_MOESM2_ESM.zip › Figure S2.jpg]

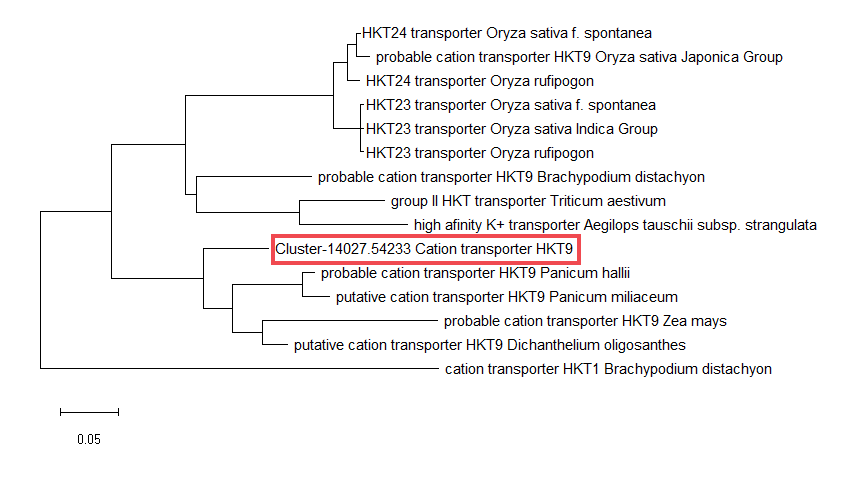


**Figure S6a**


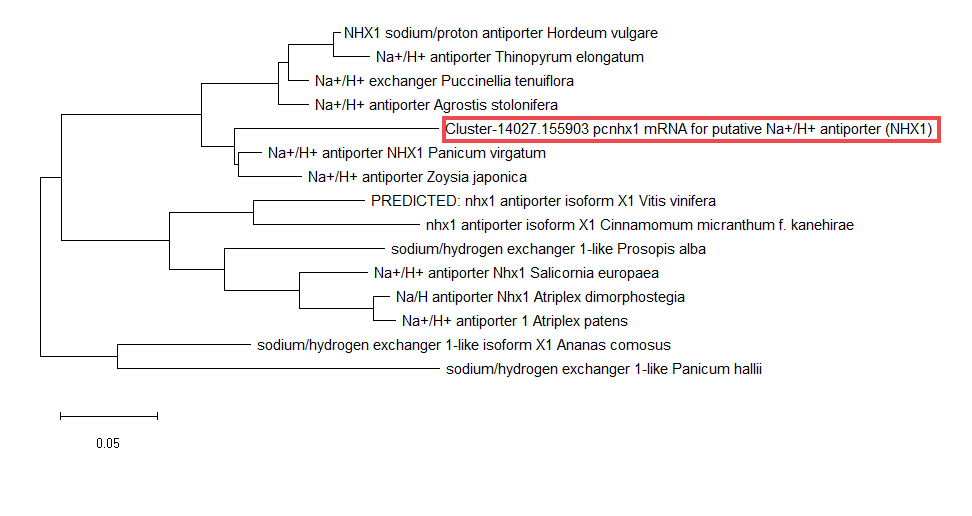


**Figure S6b**


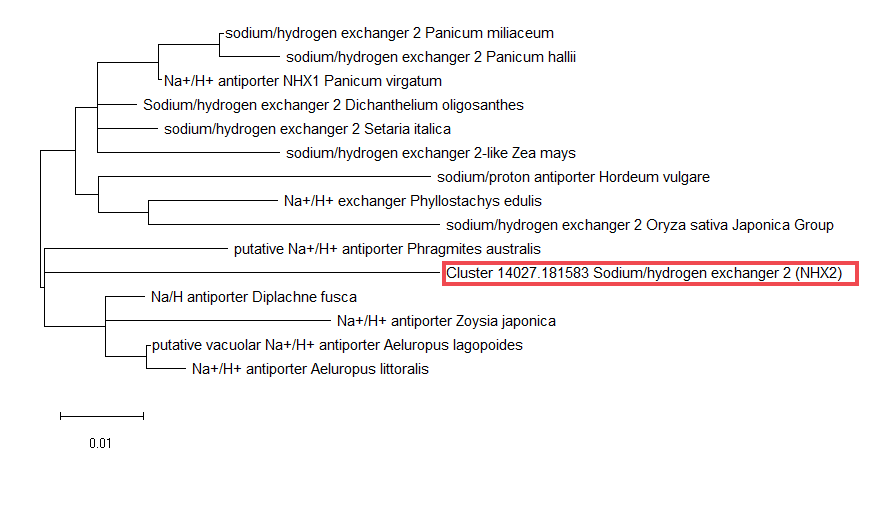


**Figure S6c**


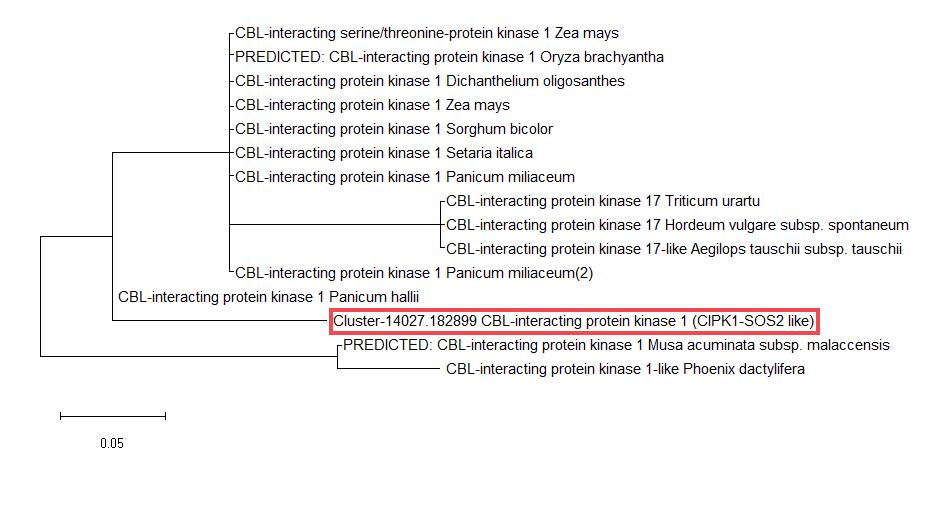


**Figure S6d**


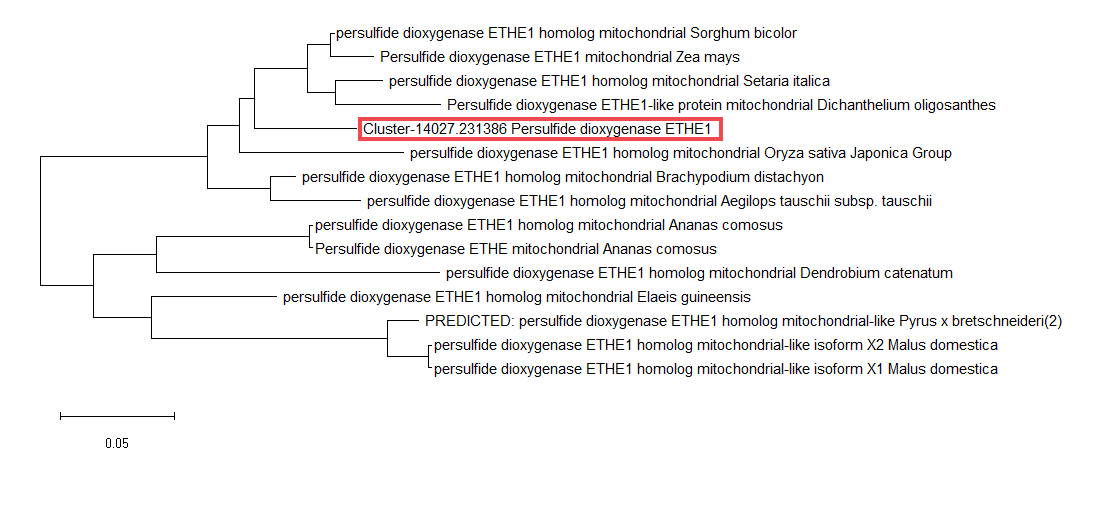


**Figure S6e**


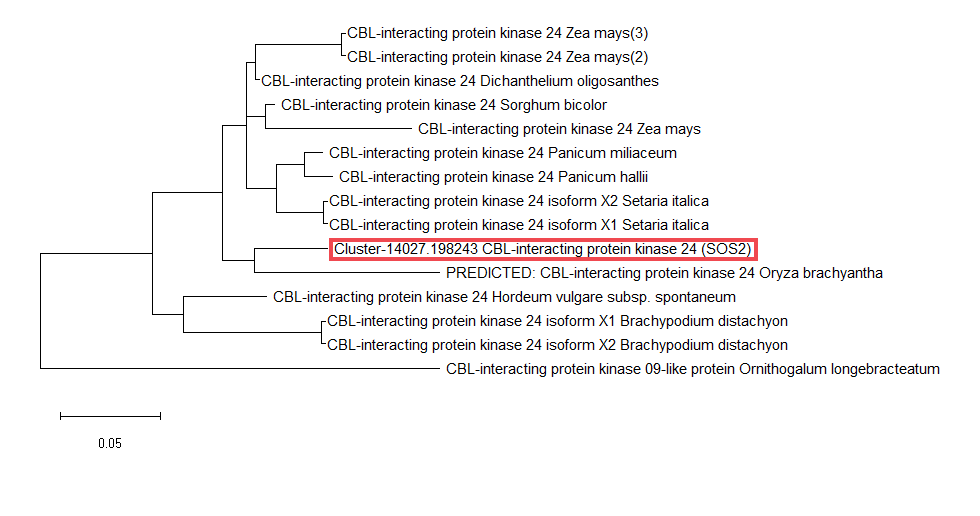


**Figure S6f**

Supplement: Supplementary file 9 — Figure S6. Phylogenetic relationship among A. donax salt responsive clusters and orthologues belonging to different plant sources. a cluster 14,027–155,903 homolog of Phramites australis Na+/H+ antiporter (NHX1). b cluster 14,027–181,583 homolog of Arabidopsis thaliana Na+/H+ exchanger 2 (NHX2). c cluster 14,027–182,899 homolog of Oryza sativa CBL-interacting protein kinase 1 (CIPK1-SOS2-like). d cluster 14,027–182,899 homolog of Oryza sativa CBL-interacting protein kinase 24 (SOS2). e cluster 14,027–54,233 homolog of Setaria italica cation transporter (HKT9). f cluster 14,027–231,386 homolog of Arabidopsis thaliana persulfide dioxygenase (ETHE1). (DOCX 171 kb) [file 12870_2019_1964_MOESM9_ESM.docx]
