# Supplementary material for: RNASeq analysis of giant cane reveals the leaf transcriptome dynamics under long-term salt stress
Source: BMC Plant Biol. 2019 Aug 15;19:355. doi: 10.1186/s12870-019-1964-y (PMC6694640; doi:10.1186/s12870-019-1964-y)
Supplement: Supplementary file 4 — Table S2. Distribution of KEGG pathways for DEGs in the three sample sets. (DOCX 15 kb) [file 12870_2019_1964_MOESM4_ESM.docx]

**Table S2 - Distribution of KEGG pathways for DEGs in the three sample sets.** Data are sorted by number of G2-S4 vs G2-CK DEGs mapping to KEGG pathways.

| ***Enriched Pathway terms*** | ***G2-S3 vs G2-CK*** | ***G2-S4 vs G2-CK*** | ***G2-S4 vs G2-S3*** | ***Total*** |
| --- | --- | --- | --- | --- |
| Carbon metabolism | 55 | 548 | 273 | 876 |
| Biosynthesis of amino acids | 44 | 463 | 203 | 710 |
| Carbon fixation in photosynthetic organisms | 37 | 259 | 149 | 445 |
| Ribosome | 7 | 252 | 148 | 407 |
| Plant hormone signal transduction | 52 | 252 | 89 | 393 |
| Starch and sucrose metabolism | 41 | 249 | 102 | 392 |
| Phenylpropanoid biosynthesis | 61 | 208 | 103 | 372 |
| Oxidative phosphorylation | 20 | 257 | 85 | 362 |
| Glycolysis / Gluconeogenesis | 26 | 237 | 83 | 346 |
| Glyoxylate and dicarboxylate metabolism | 18 | 189 | 129 | 336 |
| Pyruvate metabolism | 30 | 204 | 101 | 335 |
| Arginine and proline metabolism | 31 | 186 | 99 | 316 |
| Cysteine and methionine metabolism | 15 | 182 | 86 | 283 |
| Phenylalanine metabolism | 41 | 161 | 77 | 279 |
| Pantothenate and CoA biosynthesis | 7 | 249 | 19 | 275 |
| Glycerophospholipid metabolism | 22 | 154 | 78 | 254 |
| Peroxisome | 16 | 158 | 79 | 253 |
| Protein processing in endoplasmic reticulum | 19 | 178 | 46 | 243 |
| Alanine, aspartate and glutamate metabolism | 19 | 143 | 77 | 239 |
| Citrate cycle (TCA cycle) | 23 | 137 | 67 | 227 |
| Glutathione metabolism | 11 | 155 | 59 | 225 |
| Purine metabolism | 20 | 156 | 49 | 225 |
| Glycine, serine and threonine metabolism | 17 | 132 | 70 | 219 |
| Cyanoamino acid metabolism | 19 | 115 | 75 | 209 |
| Amino sugar and nucleotide sugar metabolism | 20 | 135 | 48 | 203 |
| AMPK signaling pathway | 20 | 119 | 54 | 193 |
| Plant-pathogen interaction | 14 | 135 | 43 | 192 |
| 2-Oxocarboxylic acid metabolism | 12 | 128 | 52 | 192 |
| Lysosome | 23 | 112 | 54 | 189 |
| Tyrosine metabolism | 7 | 114 | 59 | 180 |
| Methane metabolism | 9 | 120 | 48 | 177 |
| Pentose phosphate pathway | 5 | 114 | 53 | 172 |
| RNA degradation | 10 | 105 | 30 | 164 |
| Endocytosis | 12 | 111 | 38 | 161 |
| Fatty acid metabolism | 7 | 105 | 49 | 161 |
| Phagosome | 21 | 107 | 33 | 161 |
| RNA transport | 9 | 109 | 40 | 158 |
| Photosynthesis | - | 113 | 93 | 156 |
| Fructose and mannose metabolism | 5 | 102 | 48 | 155 |
| Phenylalanine, tyrosine and tryptophan biosynthesis | 9 | 98 | 44 | 151 |
| Ubiquinone and other terpenoid-quinone biosynthesis | 9 | 97 | 43 | 149 |
| Fatty acid degradation | 3 | 88 | 49 | 140 |
| Galactose metabolism | 13 | 92 | 28 | 133 |
| Spliceosome | 11 | 79 | 22 | 112 |
| alpha-Linolenic acid metabolism | 3 | 63 | 43 | 109 |
| Carotenoid biosynthesis | 14 | 58 | 31 | 103 |
| mRNA surveillance pathway | 5 | 70 | 26 | 101 |
| Drug metabolism - cytochrome P450 | 8 | 62 | 29 | 99 |
| Aminoacyl-tRNA biosynthesis | 3 | 78 | 14 | 95 |
| Porphyrin and chlorophyll metabolism | 2 | 61 | 22 | 85 |
